# Reactivity and Selectivity of Photocatalytic Nitrate Reduction Reaction to Ammonia: Pt, Pd, Ir, and Rh Single Atoms on p‑Type TiO_2_ Nanotubes

**DOI:** 10.1021/jacs.6c16392

**Published:** 2026-10-14

**Authors:** Hayoon Jung, Johannes Will, Michal Langer, Rostislav Langer, Štěpán Kment, Michal Otyepka, Erdmann Spiecker, Patrik Schmuki

**Affiliations:** a Department of Materials Science and Engineering, WW4-LKO, 537487 Friedrich-Alexander-Universität Erlangen-Nürnberg, Martensstraße 7, 91058 Erlangen, Germany; b Regional Centre of Advanced Technologies and Materials, Czech Advanced Technology and Research Institute (CATRIN), 48207 Palacký University Olomouc, Šlechtitelů 27, 78371 Olomouc, Czech Republic; c Institute of Micro- and Nanostructure Research & Center for Nanoanalysis and Electron Microscopy (CENEM), IZNF, 9171 Friedrich-Alexander-Universität Erlangen-Nürnberg, Cauerstraße 3, 91058 Erlangen, Germany; d IT4Innovations, VSB−Technical University of Ostrava, 17. listopadu 2172/15, 70800 Ostrava-Poruba, Czech Republic

## Abstract

We present a systematic study of noble metal (Pt, Pd, Ir, and Rh) single-atom cocatalysts decorated on p-type and quantum-confined TiO_2_ nanotubes for photocatalytic nitrate reduction to ammonia. All single-atom cocatalysts were prepared through a reactive deposition using different metal precursors, resulting in a single-atom oxidation state (δ+) of δ ≈ 2. At a similar loading concentration (0.2–0.3 at%) and in the absence of any sacrificial agent, Pd single atoms exhibit the highest element-specific activity for ammonia production, while Pt single atoms display a prominent photocatalytic activity with a strong tendency of nitrite reduction toward ammonia. Rh single atoms achieve the highest ammonia selectivity, whereas Ir single atoms predominantly produce nitrite due to inhibited subsequent reduction. Comparisons with single atoms deposited on conventional TiO_2_ (P25) and with nanoparticles supported on the nanotubes reveal that both the semiconductor properties (quantum confinement effect and p-type conductivity) and the speciation of the cocatalyst (single atom) can significantly enhance activity and selectivity. These findings aid in establishing design principles for different single-atom catalysts toward efficient and selective photocatalytic nitrogen valorization.

## Graphical abstract

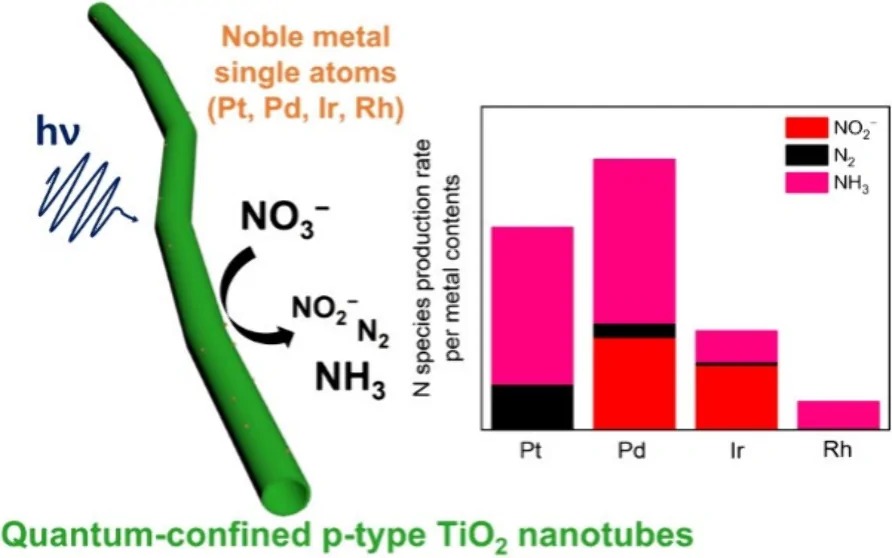

Single-atom (SA) catalysts have over the past decade attracted wide interest across various catalytic disciplines, including heterogeneous catalysis,^1^ electrocatalysis,^2^ and more recently, photocatalysis,^3,4^ due to the high utilization efficiency of noble metals as well as often unexpected reaction selectivity. In conventional photocatalysis, a range of classic crystalline semiconductors, such as TiO_2_, ZnO, and CdS, are widely used because they can efficiently harvest light upon excitation with photon energy exceeding their band gap, and provide electron–hole pairs that can readily migrate to the semiconductor surface.^5^ There, they can trigger diverse redox reactions, such as H_2_ evolution,^6–8^ CO_2_ reduction,^9^ and nitrate (NO_3_^–^) reduction reaction.^10,11^ Among these photocatalytic reactions, the reduction of nitrate to ammonia (NH_3_) has emerged as a promising strategy for wastewater reclamation and upcycling.^11–14^ However, despite extensive research on photocatalytic nitrate reduction, attaining high activity and selectivity toward ammonia remains challenging, as the reaction involves a complex multielectron transfer process and can generate several nitrogen-containing byproducts.^13–15^ Additionally, the reaction rate at many semiconductor surfaces is hindered by sluggish charge transfer from the semiconductor to reactants.^16^ To address this issue, noble metal cocatalysts are frequently employed to facilitate charge transfer and enhance the overall photocatalytic performance.^17,18^ Given the high cost of noble metals, SA cocatalysts have come into the spotlight as a pioneering solution due to their maximized atom utilization.^19–22^ Moreover, SA cocatalysts can form strong interactions with the semiconductor substrate, often surpassing conventional scaling relations and delivering an exceptional catalytic activity.^22,23^

Noteworthy in the context of the present work is also that most studies on photocatalytic nitrate reduction reactions rely on the use of hole scavengers (e.g., formic acid, humic acid, and methanol), which are difficult to implement under practical conditions.^11,24–28^ Especially for TiO_2_, nitrate reduction to ammonia is only possible in the presence of hole scavengers that allow the generation of an aqueous CO_2_ radical anion (i.e., CO_2_^•–^) by hole-induced oxidation—the direct reduction of nitrate to ammonia is thermodynamically impossible with the conduction band level of classic TiO_2_ polymorphs.^13,29^ However, thin-walled p-type TiO_2_ nanotubes (p-TiO_2_ NTs), which show a significant quantum confinement effect resulting in an elevated conduction band minimum and exhibit promoted hole mobility facilitating sluggish counter-reaction of water oxidation, have recently been shown to be an effective platform for photocatalytic ammonia production with plain nitrate aqueous solution.^30^ In addition, the combination of metal SA cocatalysts with various semiconductors has been reported to yield photocatalytic nitrate reduction and ammonia production,^27,28^ even in the absence of hole scavengers.^30–32^ Nonetheless, the exploration of SA cocatalysts for photocatalytic nitrate reduction remains largely limited despite their significant potential, particularly when compared to the well-established studies on photocatalytic hydrogen evolution reaction^33,34^ or electrocatalytic oxygen reduction reaction.^35,36^

Herein, we systematically investigate different SA cocatalysts (Pt, Pd, Ir, and Rh SAs) anchored on p-TiO_2_ NTs for photocatalytic nitrate reduction to ammonia. Notably, while Pd SAs outperform the others for photocatalytic ammonia production, each metal SA displays distinct activity and selectivity profiles, highlighting a strong element-specific behavior. Pt SAs show both prominent photocatalytic activity and selectivity toward ammonia; Pd SAs achieve the highest ammonia production rate but produce nitrite (NO_2_^–^) as a major side product, which is consistent with their (thermo)­catalytic characteristics. Ir SAs mainly generate nitrite due to suppressed subsequent reduction whereas Rh SAs exhibit the highest ammonia selectivity, aligning well with their electrochemical nitrate/nitrite reduction behavior. In comparison with metal SAs on a conventional TiO_2_ substrate (i.e., commercial P25) and metal nanoparticles (NPs) supported on p-TiO_2_ NTs, single atoms on p-TiO_2_ NTs (metal SAs/p-TiO_2_ NTs) clearly exhibit superior performance, highlighting the combined importance of the semiconductor’s electronic properties and the metal cocatalysts’ speciation.

We first prepared p-TiO_2_ NTs as a suitable semiconductor substrate via a hydrothermal method,^30,37^ as described in the Supporting Information (SI). In contrast to the classic TiO_2_ materials, these nanotubes enable direct photocatalytic nitrate reduction to ammonia without the assistance of hole scavengers due to the band gap widening induced by quantum confinement.^30^ The scanning electron microscopy (SEM) image in Figure 1a shows the tubular morphology with a length of several micrometers and an outer diameter of approximately 10 nm. The p-TiO_2_ NTs have a TiO_2_(B) monoclinic crystal structure, exhibiting an X-ray diffraction (XRD) pattern (Figure 1b) with diffraction planes of the TiO_2_(B) phase (ICDD 046-1237). The quantum confinement effect in p-TiO_2_ NTs is evident from incident-photon-to-current efficiency (IPCE) measurements and the corresponding Tauc plot (Figure S1a), where a band gap at approximately 3.5 eV is obtained, which is significantly larger than that of TiO_2_ bulk materials (3.0–3.2 eV). Furthermore, the negative slope in Mott–Schottky plots (Figure S1b) evidently confirms the p-type conductivity of the NTs. Based on the flat-band potential from the Mott–Schottky plots and the previously determined band gap, the conduction band position of p-TiO_2_ NTs is estimated to be at −0.7 V vs. NHE at pH 7, which is more negative than that of the conventional TiO_2_ materials, thus enabling more reduction reactions at more negative redox potentials.^30,38^

**Figure 1:**
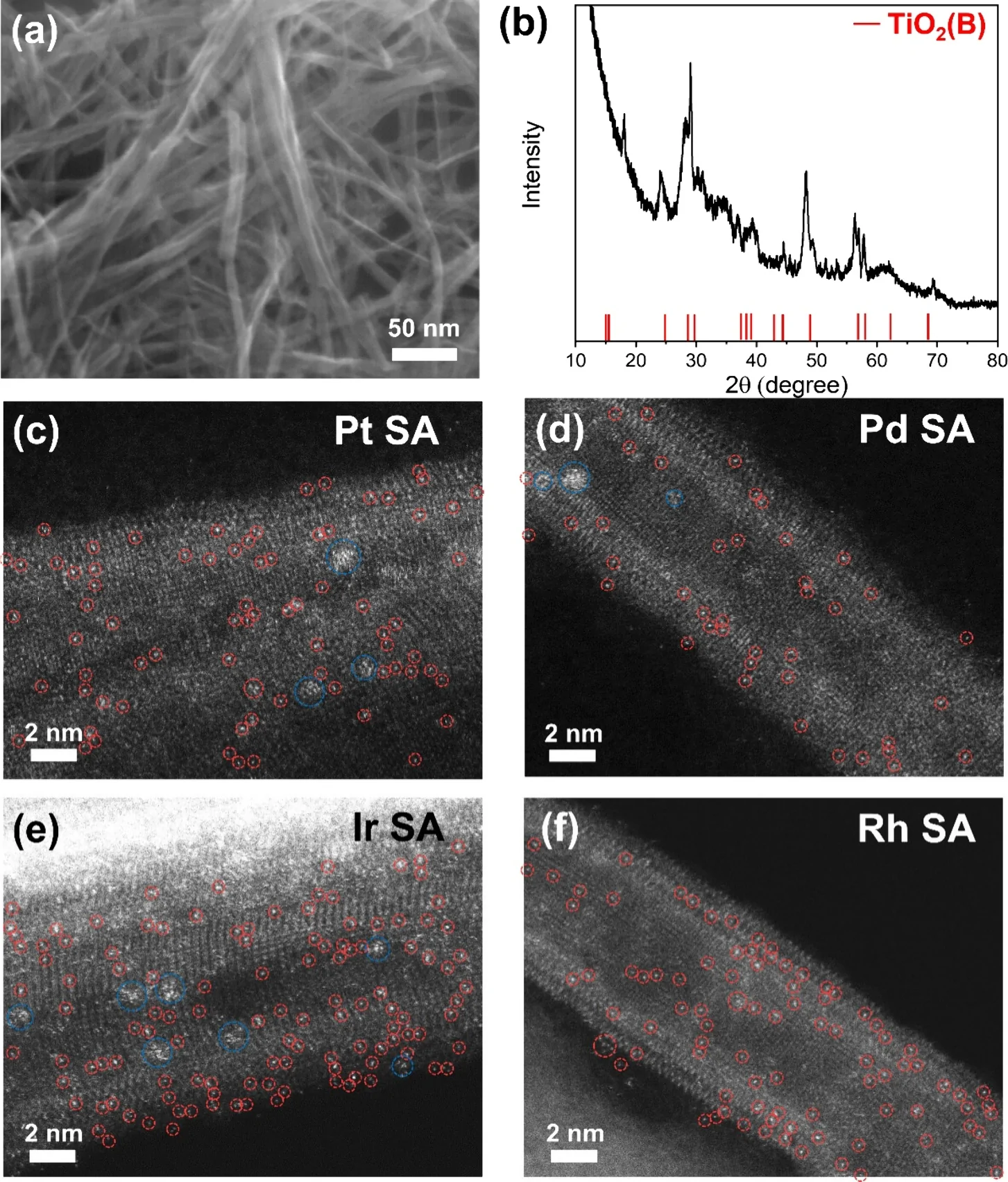
(a) SEM and (b) XRD pattern of p-TiO_2_ NTs. The reference positions were taken from the ICDD database. (c–f) HAADF-STEM image with highlighted SAs (red circles) and rafts (blue circles) of (c) Pt SAs/p-TiO_2_ NTs, (d) Pd SAs/p-TiO_2_ NTs, (e) Ir SAs/p-TiO_2_ NTs, and (f) Rh SAs/p-TiO_2_ NTs.

We then loaded metal (Pt, Pd, Ir, or Rh) SAs on the p-TiO_2_ NTs via a reactive deposition approach (see the SI for experimental details).^20–22,39^ As revealed by SEM images in Figure S2, the p-TiO_2_ NTs retained their original morphology after each metal SA decoration, without the formation of any discernible NPs. The presence of metal SAs is confirmed by high-angle annular dark-field scanning transmission electron microscopy (HAADF-STEM) images in Figure 1c–f. In all cases, the SAs are uniformly distributed across the NT surface with a surface density of approximately (3–5) × 10^5^ μm^–2^—only a few slightly assembled 2D SAs-rafts are observed (Figure S3).

X-ray photoelectron spectroscopy (XPS) for the corresponding core-level regions (Pt 4f, Pd 3d, Ir 4f, and Rh 3d) is shown in Figure 2. For Pt SAs/p-TiO_2_ NTs, the XPS spectrum displays characteristic 4f_7/2_ and 4f_5/2_ doublet peaks, indicating a positively charged Pt^δ+^ species with a formal charge (δ) of around δ ≈ 2. Likewise, Pd SAs/p-TiO_2_ NTs exhibit Pd 3d doublets of 3d_5/2_ peak at 337.3 eV and 3d_3/2_ peak at 342.5 eV. Ir SAs/p-TiO_2_ NTs at 61.7 eV (4f_7/2_) and 64.9 eV (4f_5/2_), and Rh SAs/p-TiO_2_ NTs at 308.8 eV (3d_5/2_) and 313.4 eV (3d_3/2_) show 4f doublet peaks and 3d doublet peaks, respectively. In every case, the peak positions are consistent with the δ+ oxidation states (δ ≈ 2), which align with previous literature for oxygen-coordinated SAs.^20–22,39–41^ Moreover, the observed XPS binding energies across all metal SAs/p-TiO_2_ NTs are well in line with the typical results for SAs prepared by reactive deposition, where metal precursors (H_2_Pt^IV^Cl_6_, (NH_4_)_2_Pd^IV^Cl_6_, Ir^III^Cl_3_, and Rh^III^Cl_3_) are reduced by Ti^3+^ at native oxygen vacancies through a galvanic replacement reaction and subsequently anchored on the TiO_2_ surface.^39,40^ In addition, it should be noted that all SA-loaded samples were adjusted to exhibit comparable metal concentrations in the range of 0.2–0.3 at%, as listed in Table S1.

**Figure 2:**
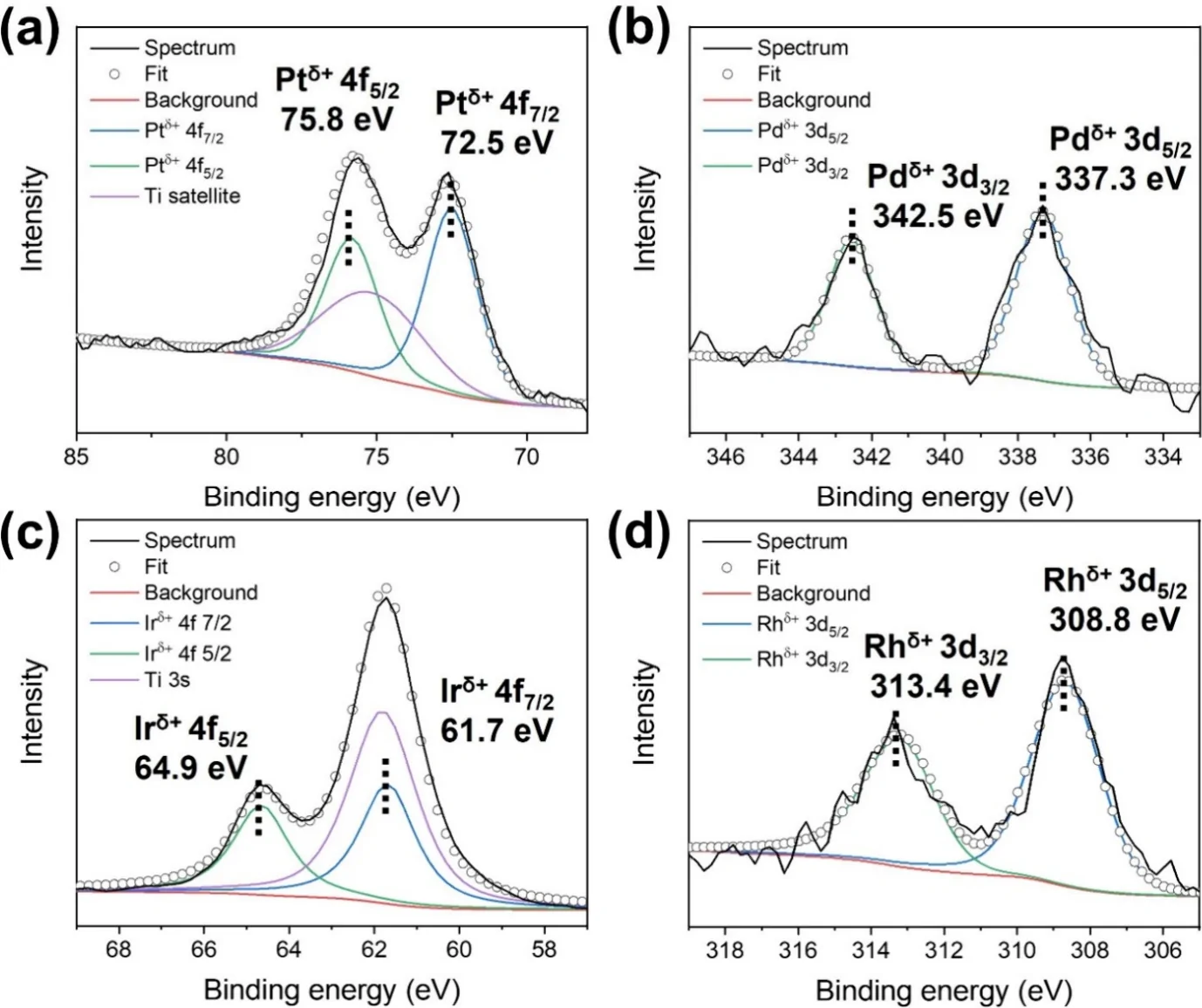
(a) Pt 4f, (b) Pd 3d, (c) Ir 4f, and (d) Rh 3d XPS spectra of (a) Pt SAs/p-TiO_2_ NTs, (b) Pd SAs/p-TiO_2_ NTs, (c) Ir SAs/p-TiO_2_ NTs, and (d) Rh SAs/p-TiO_2_ NTs, respectively.

To investigate the role of each metal SA in photocatalytic nitrate reduction to ammonia (NO_3_^–^ + 9H^+^ + 8e^–^ → NH_3_ + 3H_2_O), all metal SAs/p-TiO_2_ NTs were evaluated for this reaction in plain KNO_3_ aqueous solution under 275 nm LED irradiation. Please note that all experiments were conducted in the absence of any hole scavengers and at nitrate concentrations in the tens-of-ppm range (1 mM), reflecting conditions relevant to practical applications. Figure 3a presents the amounts of ammonia produced from the metal SAs/p-TiO_2_ NTs over 8 h, and the corresponding ammonia production rates, normalized to the respective metal concentrations, are depicted in Figure 3b (rates before metal normalization provided in Figure S4). Notably, Pd SAs deliver an ammonia production rate comparable to Pt SAs/p-TiO_2_ NTs, and surpass all other metal SAs when normalized to metal concentration. Interestingly, this trend (Pd SA > Pt SA) is consistent with prior computational results of photocatalytic reactivity^42^ and experimental studies on nitrate reduction/hydrogenation with Pt and Pd species under photocatalytic^43^ and thermocatalytic conditions.^44,45^ We also monitored the pH change of the reaction medium, as summarized in Table S2.

**Figure 3:**
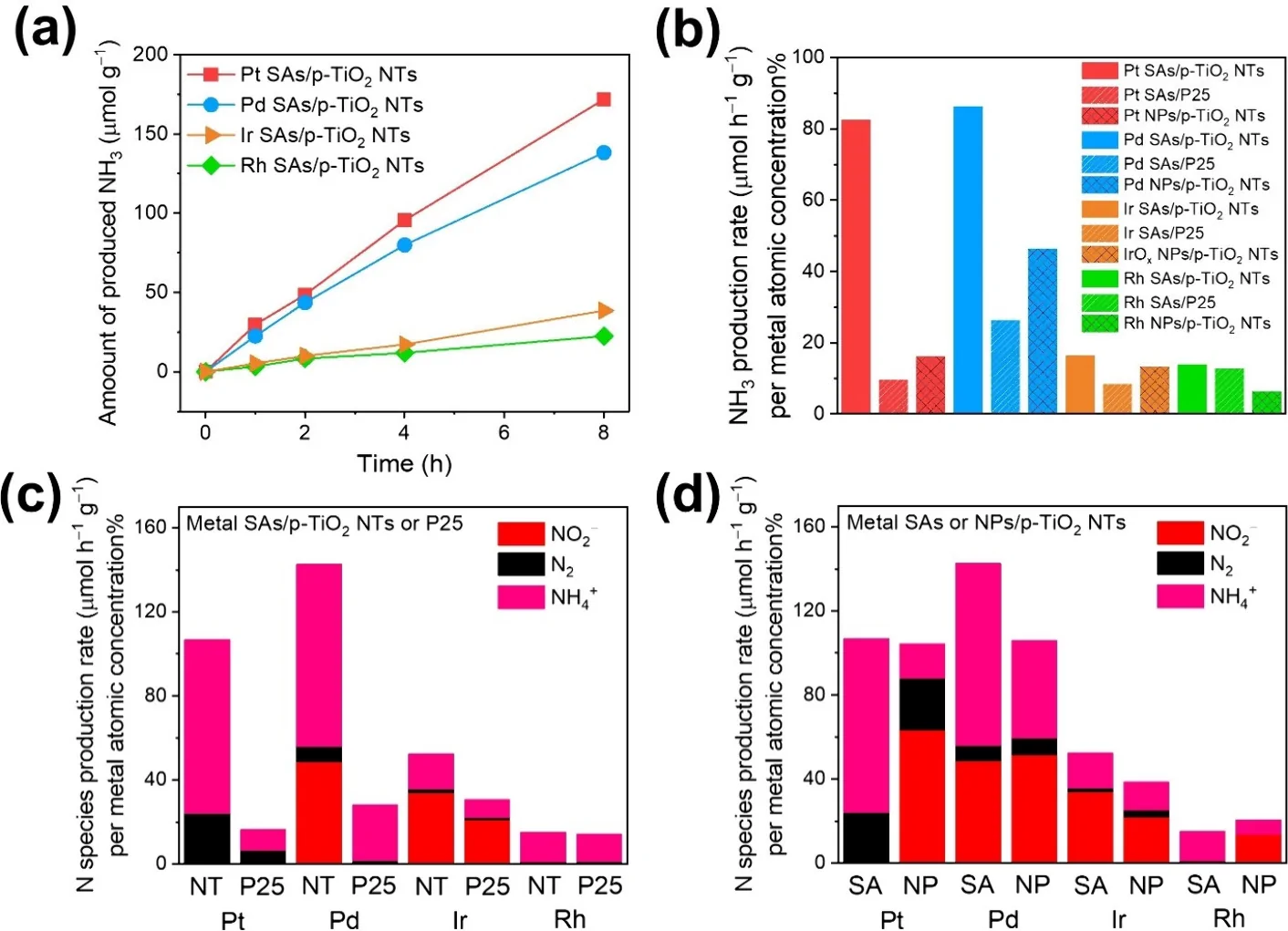
(a) Time-course ammonia production of Pt SAs/p-TiO_2_ NTs, Pd SAs/p-TiO_2_ NTs, Ir SAs/p-TiO_2_ NTs, and Rh SAs/p-TiO_2_ NTs under 275 nm LED irradiation. (b) Metal concentration-normalized ammonia production rates from nitrate reduction over various catalysts for 8 h photocatalysis. (c,d) Resultant nitrogen species after photocatalytic nitrate reduction of metal SAs/p-TiO_2_ NTs with metal concentration normalization, compared with (c) metal SAs/P25 and (d) metal NPs/p-TiO_2_ NTs.

For comparison, we investigated the same metal SAs supported on the classic benchmark TiO_2_ material, P25, which has a band gap of 3.0–3.2 eV and n-type conductivity (metal concentrations summarized in Table S3).^46^ As shown in Figure 3b, metal SAs/p-TiO_2_ NTs exhibit superior performance compared to the corresponding metal SAs/P25, highlighting the advantageous combination of quantum confinement and p-type conductivity in the NT substrates.^30,32^ A particularly important distinction lies in the quantum confinement effect: p-TiO_2_ NTs and P25 demonstrate fundamentally different capabilities for photocatalytic nitrate reduction to ammonia, as the conduction band level of conventional TiO_2_ is thermodynamically insufficient to drive this reaction.^13,29^ The pristine p-TiO_2_ NTs can generate ammonia (Figure S5a) owing to their elevated conduction band minimum, which is more negative than the reduction potential of nitrate to ammonia (−0.58 V vs. NHE at pH 7). In contrast, P25 without metal cocatalysts cannot produce detectable amounts of ammonia even after 24 h light irradiation (Figure S5b), which is consistent with prior reports.^24,28^ Another notable point is that Pd SAs also outperform the other metal SAs when deposited on P25, further supporting their higher reactivity from previous literature.^43–45^ In addition, metal NPs/p-TiO_2_ NTs (Figures S6–S9; metal concentrations provided in Table S3) were prepared for comparison, exhibiting lower metal-normalized ammonia production than the corresponding metal SAs. Furthermore, to assess the impact of metal SA loading on photocatalytic performance, ammonia production rates were evaluated for p-TiO_2_ NTs decorated with each metal SA at various loadings (Table S1). When normalized to the metal content, the most remarkable performance is achieved at a metal loading of approximately 0.2–0.3 at% (Figure S10).

It is crucial to examine other nitrogen-containing reaction products that may form during the multistep reduction of nitrate, particularly nitrite (NO_2_^–^) and nitrogen gas (N_2_), which represent the predominant side products of the reaction. Therefore, the photocatalytic formation rates of each nitrogen species were evaluated for metal SAs/p-TiO_2_ NTs, and compared with those for the corresponding SAs/P25 (Figure 3c and S11) and NPs/p-TiO_2_ NTs (Figure 3d and Figure S12). The ammonia production rates and selectivities to ammonia are summarized in Table S4. Pt SAs/p-TiO_2_ NTs predominantly produce ammonia, with N_2_ as the only side product. Similarly, ammonia is the major product for Pd SAs/p-TiO_2_ NTs; however, nitrite emerges as the second most abundant side product. Considering that the pristine p-TiO_2_ NTs generate nitrite as a major product (Figure S5), Pt and Pd SAs—particularly Pt—enhance not only the overall nitrate reduction rate but also the subsequent reduction and hydrogenation of nitrite to ammonia.^30,32,47,48^ In addition, the stronger nitrite consumption and reduction trend observed for Pt SAs distinctly resembles the established (thermo)­catalytic behavior, where Pt is more favorable in nitrite-to-ammonia conversion while Pd is more capable of overall reduction.^44,45^ Rh SAs/p-TiO_2_ NTs, despite the lowest overall activity, achieve the highest selectivity toward ammonia, consistent with their higher affinity for nitrogen intermediate adsorption^49–51^ and the reported higher efficiency for the successive nitrite reduction to ammonia^50–52^ in electrochemical nitrate reduction. In contrast, Ir SAs/p-TiO_2_ NTs mostly yield nitrite for their photocatalytic nitrate reduction. This indicates a strong inhibition of the further nitrite-to-ammonia reduction in agreement with the low intrinsic nitrite reduction activity and high NO_*x*_ poisoning susceptibility of Ir.^53–55^ We also conducted the same photocatalytic reactions with nitrite instead of nitrate, and the resulting activity and selectivity trends are consistent with those observed for nitrate reduction (Figure S13). Furthermore, we examined the H_2_ evolution, a representative competing reaction during the photocatalytic nitrate reduction (Figure S14). Apparently, all metal SAs/p-TiO_2_ NTs show relatively minimal H_2_ production compared to ammonia. Regarding the counter water-oxidation reaction, the accumulation of free O_2_ was hardly observed; however, we identified surface peroxo complexes (Figure S15) and peroxide species after photocatalysis (Figure S16). Density functional theory (DFT)+U calculations were performed for Pt, Pd, Rh, and Ir SA models on p-TiO_2_ NTs to compare the reaction thermodynamics of their nitrate reduction (Figures S17 and S18). Nitrate adsorption on the metal site and the reduction sequence from *NO_3_ to *NO are exergonic on all four systems. After the formation of *NO, N protonation to *NHO is thermodynamically more favorable than O protonation to *NOH for every metal (see the Supporting Information (SI) for details).

Replacing the semiconductor substrate with P25 alters the selectivity of metal SAs with a clear decrease in the overall photocatalytic nitrate conversion (Figure 3c and Figure S11). Interestingly, while the selectivity toward ammonia is slightly reduced for Pt SAs, there is a substantial increase in ammonia selectivity for Pd SAs, without any nitrite formation. Such changes in selectivity and product distributions likely arise from the intrinsic difference between the pristine p-TiO_2_ NTs, which can generate all major nitrogen-containing products, and P25, which is incapable of producing any detectable amounts of these products (Figure S5).^24^ Furthermore, the Pt, Pd, and Rh SAs investigated here show clear advantages in selectivity (higher NH_3_ selectivity) and product distribution (no nitrite formation in Pt and Rh SAs) compared to their NP counterparts, except in the case of Ir (Figure 3d and Figure S12). This highlights that not only the semiconductor properties but also the atomic dispersion state of the cocatalyst critically governs both activity and selectivity in photocatalytic nitrate reduction.

In summary, this work provides a systematic and comprehensive comparison of different metal SA cocatalysts (Pt, Pd, Ir, and Rh) on p-TiO_2_ NTs with quantum confinement effects for photocatalytic nitrate reduction to ammonia without hole scavengers. All SAs were prepared via the reactive deposition method to achieve comparable oxidation states (δ^+^, δ ≈ 2) and metal loadings (0.2–0.3 at%), allowing a direct evaluation of element-specific activity and selectivity. In particular, Pd SAs exhibit the highest metal-normalized activity, whereas Pt SAs combine high activity with strong ammonia selectivity. Rh SAs excel in nitrite-to-ammonia conversion with their highest ammonia selectivity, while Ir SAs mostly yield nitrite due to suppressed subsequent reduction. Moreover, all SAs on p-TiO_2_ NTs outperform their counterparts on P25 and NP-based systems, indicating the decisive influence of both semiconductor properties and cocatalyst dispersion state on photocatalytic performance.

## Supplementary Material



## Notes

Experimental section; metal atomic concentrations of metal SAs/p-TiO_2_ NTs; pH change of reaction media for metal SAs/p-TiO_2_ NTs after photocatalysis; metal atomic concentrations of metal SAs/P25 and metal NPs/p-TiO_2_ NTs; ammonia production rates and selectivities to ammonia of various catalysts; Tauc plot and Mott–Schottky plots of p-TiO_2_ NTs; SEM images of Pt SAs/p-TiO_2_ NTs, Pd SAs/p-TiO_2_ NTs, Ir SAs/p-TiO_2_ NTs, Rh SAs/p-TiO_2_ NTs; statistical distributions of SAs and rafts; ammonia production rates over various catalysts; SEM image and Pt 4f XPS spectrum of Pt NPs/p-TiO_2_ NTs; SEM image and Pd 3d XPS spectrum of Pd NPs/p-TiO_2_ NTs; SEM image and Ir 4f XPS spectrum of IrO_*x*_ NPs/p-TiO_2_ NTs; SEM image and Rh 3d XPS spectrum of Rh NPs/p-TiO_2_ NTs; ammonia production rates of metal SAs/p-TiO_2_ NTs with different metal concentrations; nitrogen species production rates of metal SAs/p-TiO_2_ NTs and metal SAs/P25; nitrogen species production rates of metal SAs/p-TiO_2_ NTs and metal NPs/p-TiO_2_ NTs; nitrogen species production rates of p-TiO_2_ NTs and P25; nitrogen species production rates of metal SAs/p-TiO_2_ NTs from nitrite reduction; H_2_ production rates of metal SAs/p-TiO_2_ NTs during nitrate reduction; O 1s XPS spectra of metal SAs/p-TiO_2_ NTs before and after photocatalysis; H_2_O_2_ detection by WaterWorks test strip and spectrophotometry with potassium titanium oxalate; DFT+U-calculated Gibbs free-energy profiles for nitrate reduction reaction to ammonia; optimized structures of the reaction intermediates on metal SA/p-TiO_2_ NT models (PDF)
