# Supplementary material for: Reactivity and Selectivity of Photocatalytic Nitrate Reduction Reaction to Ammonia: Pt, Pd, Ir, and Rh Single Atoms on p‑Type TiO2 Nanotubes
Source: J Am Chem Soc. 2026 Oct 14;148(42):45301–7. doi: 10.1021/jacs.6c16392 (PMC13721143; doi:10.1021/jacs.6c16392)
Supplement: Supplementary file 1 [file ja6c16392_si_001.pdf]

Supporting information

Reactivity and Selectivity of Photocatalytic Nitrate Reduction Reaction to Ammonia:

Pt, Pd, Ir, and Rh Single Atoms on p-Type TiO_2 Nanotubes

Hayoon Jung, [a,b] Johannes Will, [c] Michal Langer, [d] Rostislav Langer, [d] Štěpán Kment, [b] Michal Otyepka, [b,d] Erdmann Spiecker, [c] and Patrik Schmuki [a,b]*

[a] Department of Materials Science and Engineering, WW4-LKO, Friedrich-Alexander-Universität Erlangen-Nürnberg, Martensstraße 7, 91058 Erlangen, Germany

[b] Regional Centre of Advanced Technologies and Materials, Czech Advanced Technology and Research Institute (CATRIN), Palacký University Olomouc, Šlechtitelů 27, 78371 Olomouc, Czech Republic

[c] Institute of Micro- and Nanostructure Research & Center for Nanoanalysis and Electron Microscopy (CENEM), IZNF, Friedrich-Alexander-Universität Erlangen-Nürnberg, Cauerstraße 3, 91058 Erlangen, Germany

[d] IT4Innovations, VSB – Technical University of Ostrava, 17. listopadu 2172/15, 70800 Ostrava-Poruba, Czech Republic

E-mail: schmuki@ww.uni-erlangen.de

Experimental Section

Chemicals and Materials

TiO_2 anatase nanopowder (Sigma–Aldrich, 99.7%), NaOH (Carl Roth, ≥99%), HCl (Carl Roth, 37%), Na_2SO_4 (Carl Roth, ≥99%), $H_2PtCl_6·6H_2O$ (Metakem, 40.17% metallic Pt weight concentration), $(NH_4)_2PdCl_6$ (Sigma–Aldrich, 99.95%), $IrCl_3·3H_2O$ (Thermo Fisher Scientific, 99.8% metals basis), $RhCl_3·3H_2O$ (Thermo Fisher Scientific, 99.99% metals basis), P25 (Sigma–Aldrich, ≥99.5%), KNO_3 (Sigma–Aldrich, ≥99.0%), KNO_2 (Fluka, ≥98%), potassium titanium oxalate (Sigma–Aldrich, ≥90%, Ti basis), H_2O_2 (Carl Roth, 30%), and H_2SO_4 (Carl Roth, 96%) were used as received without any purification. Highly purified water with a resistivity exceeding 18.2 MΩ·cm was utilized in aqueous solutions.

Synthesis of p-TiO_2 NTs

p-TiO_2 NTs were synthesized via a hydrothermal method. Specifically, 3.0 g of anatase-phase TiO_2 nanopowder was dispersed in 40 mL of 10 M NaOH aqueous solution. The mixture was then transferred to a 100 mL Teflon-lined autoclave and heated at 150 °C for 48 h. After the reaction, the resulting product was sequentially washed with purified water and 0.1 M HCl aqueous solution, followed by drying at 70 °C for subsequent use.

Metal SAs and NPs Deposition

To prepare metal (Pt, Pd, Ir, or Rh) SAs on TiO_2 substrates (p-TiO_2 NTs and P25), 40 mg of each TiO_2 sample was first dispersed in 200 mL of deionized water under continuous stirring

to obtain a homogeneous suspension. The suspension was then purged with Ar for 15 min to remove dissolved oxygen. Subsequently, the corresponding metal precursor, H_2PtCl_6 for Pt, $(NH_4)_2PdCl_6$ for Pd, $IrCl_3$ for Ir, or $RhCl_3$ for Rh, was added to the suspension to give final precursor concentrations of 0.05, 0.01, 0.1, and 0.01 mM, respectively. After precursor addition, the suspension was continuously stirred in the dark for 1 h to allow deposition of the metal species onto the TiO_2 surface. The resulting solid was then separated from the suspension by centrifugation at 4,000 rpm for 20 min. To systematically control the loading of the individual metal SAs, the precursor concentration was further varied for each metal while keeping the other preparation conditions unchanged, as summarized in Table S1. For the photodeposition of metal NPs, 20 mg of p-TiO_2 NTs was suspended in 20 mL of a methanol–water mixture (50 vol%). After Ar purging for 15 min, H_2PtCl_6, $(NH_4)_2PdCl_6$, $IrCl_3$, or $RhCl_3$ was added to reach a precursor concentration of 0.05, 0.05, 0.5, or 0.05 mM, respectively. The suspension was then irradiated with UV LED light (λ = 275 nm, power density = 20 mW cm^{-2}) for 24 h. The final product was washed by centrifugation at 4,000 rpm for 20 min.

Characterization

SEM images were acquired using a Hitachi S-4800 instrument. HAADF-STEM was performed using a high-resolution transmission electron microscope (Spectra 200 C-FEG, Thermo Fisher Scientific), equipped with probe aberration correction and a cold field emission gun (X-CFEG). An XRD pattern of p-TiO_2 NTs was recorded using a Philips X'Pert MPD diffractometer (Panalytical X'celerator detector) with graphite-monochromatized Cu Kα radiation (λ =

0.15406 nm). XPS was conducted with a PHI 5600 spectrometer (USA), utilizing monochromatic Al Kα radiation (1486.6 eV, 300 W), to examine the chemical composition of the samples. The pH of reaction media was evaluated by a SevenCompact Duo S213 pH/conductivity meter (Mettler Toledo). (Photo)electrochemical analyses, including IPCE measurements for band gap analysis and Mott-Schottky analyses, were carried out using a standard three-electrode setup. The configuration consisted of p-TiO_2 NTs as the working electrode, a platinum plate as the counter electrode, and an Ag/AgCl electrode as the reference. The electrolyte was a 0.1 M Na_2SO_4 aqueous solution purged with Ar. IPCE measurements were performed under a constant bias of −0.5 V (vs. Ag/AgCl) across a wavelength range of 250–600 nm using a 150 W Xe lamp (Oriel 6365) coupled with an Oriel Cornerstone 7400 1/8 m monochromator. In the Mott-Schottky analyses, capacitance was evaluated based on electrochemical impedance data obtained at various applied potentials. The measurements were conducted by superimposing a 10 mV AC perturbation over a frequency of 500, 1000, and 2000 Hz. H_2O_2 formation after 24 h of photocatalysis was examined using WaterWorks™ Low Range Peroxide Test Strips (Industrial Test Systems, Inc. , Part No. 481015) and quantitatively determined by potassium titanium oxalate colorimetry. 1 mL of the reaction solution with the catalyst filtration or the freshly prepared H_2O_2 solution with various concentrations was mixed with potassium titanium oxalate reagent (50 g L^{-1}, 0.1 mL) and H_2SO_4 solution (1 M, 0.05 mL) for 15 min, and the absorbance of the resulting titanium–peroxide complex was measured at 400 nm.

Photocatalysis

Photocatalytic nitrate or nitrite reduction was carried out under UV LED light irradiation (λ = 275 nm, power density = 10 mW cm^{-2}). A total of 2.0 mg of catalyst was dispersed in 10 mL of 1 mM KNO_3 or KNO_2 aqueous solution within a quartz reaction cell. Prior to illumination, the solution was purged with Ar for 15 min to establish anaerobic conditions. The concentrations of nitrogen-containing products were determined using ion chromatography (833 Basic IC plus and 930 Compact IC Flex, Metrohm). The amount of N_2 produced was estimated based on the nitrogen mass balance of the reactant and other products. The amount of H_2 was determined by a gas chromatograph (GCMS-QP2010SE, SHIMADZU) with a thermal conductivity detector.

DFT+U Calculation

The TiO_2(B)(100) slab for p-TiO_2 NTs was constructed from the optimized coordinates for low-index TiO_2(B) surfaces.[S1] Their periodic models provide an established atomistic description of the crystallographic surface while retaining the characteristic octahedral and square-pyramidal Ti environments of the TiO_2(B) phase. The 7.51 × 13.25 Å^2 surface cell contained 48 Ti and 96 O atoms, and a 31 Å lattice vector was used perpendicular to the surface. The periodic model was designed to describe the local environment of the catalytic center rather than the complete mesoscale morphology of the nanotube. This is a reasonable local representation because the experimental nanotubes have an outer diameter of approximately 10

nm, substantially larger than the dimensions of the vacancy-bound single-atom site and its adsorbates.

A surface O atom was removed to generate an oxygen-vacancy anchoring site, at which one Pt, Pd, Rh, or Ir atom was introduced. The exposed undercoordinated Ti centers were terminated with surface hydroxyl groups to represent the hydrated surface environment.

The defective hydroxylated slabs containing the vacancy-bound metal atom were first fully relaxed. For the subsequent optimization of reaction intermediates, the upper two surface layers, the surface hydroxyl groups, the metal atom, and the adsorbate were allowed to relax, while the deeper slab region was held fixed at the geometry obtained from the initial slab optimization. Several initial configurations of the vacancy-bound metal atom and adsorbed nitrate were examined for each metal. The lowest-energy stable nitrate-bound configuration obtained within this screening was used to initiate the corresponding reaction pathway. Because adsorption and hydroxylation occur on only one side of the slab, dipole corrections were applied along the surface-normal direction. All periodic calculations employed charge-neutral simulation cells.

Spin-polarized, ground-state density functional theory calculations were performed using the Vienna *Ab initio* Simulation Package (VASP) within the projector augmented-wave formalism.[S2–S4] The exchange-correlation energy was described using the Perdew-Burke-Ernzerhof generalized-gradient approximation, and a plane-wave cutoff energy of 520 eV was used.[S5] Long-range dispersion interactions were included using the DFT-D3 method with Becke-Johnson damping.[S6,S7] The electronic self-consistency criterion was 10^{-5} eV, and

atomic positions were relaxed until the residual forces were below 0.03 eV Å^{-1}. The lattice vectors were held fixed during the structural optimizations. Gaussian smearing with a width of 0.03 eV was applied, and the Brillouin zone was sampled using a Γ-centered 3×3×1 k-point mesh. Nonspherical gradient corrections inside the PAW spheres were included, and symmetry was disabled for the defective and adsorbate-containing structures. The on-site Coulomb interaction of the Ti 3d states was described using the rotationally invariant Dudarev DFT+U formalism with U_{eff} of 4.2 eV.[S8,S9]

Vibrational frequency calculations were used to obtain zero-point-energy and finite-temperature contributions to the free energies. For surface intermediates, the metal single atom and the atoms of the adsorbed intermediate were displaced, while the TiO_2(B) support was kept fixed. The thermochemical contributions were evaluated at 298.15 K using VASPKIT.[S10] For adsorbed intermediates, vibrational contributions were included, whereas the separately optimized gas-phase reference species were treated using the corresponding translational, rotational, vibrational, and electronic contributions.

Proton-coupled electron-transfer steps were described using the computational hydrogen electrode:[S11]

$$\mu(\mathrm{H}^+ + e^-) = \frac{1}{2}G(\mathrm{H}_2) - eU.$$

The profiles were presented relative to adsorbed $^*NO_3$, whose free energy was set to zero. For Ir, a stable $^*NO_2H$ intermediate was not obtained, and the corresponding part of the pathway was therefore represented by the balanced net reaction $^*NO_2 + H_2 \rightarrow {}^*NO + H_2O$.

The nitrate adsorption free energy (computed at T = 298 K) was calculated using the charge-neutral HNO_3/H_2 reference:

$$\Delta G_{\mathrm{ads}}(\mathrm{NO_3}) = G(*\,\mathrm{NO_3}) - G(*) - G\big(\mathrm{HNO_3(g)}\big) + \frac{1}{2}G\big(\mathrm{H_2\,(g)}\big).$$

Molecular-water adsorption was evaluated as:

$$\Delta G_{\mathrm{ads}}(\mathrm{H_2O}) = G(*\,\mathrm{H_2O}) - G(*) - G\big(\mathrm{H_2O(g)}\big).$$

The thermodynamic potential-determining step (PDS) was identified as the proton-coupled electron-transfer step with the largest $\Delta G/n_e$ value, where n_e is the number of electrons transferred in the corresponding step.

Table S1. Metal atomic concentrations of metal SAs/p-TiO_2 NTs, determined by XPS measurements. The metal precursor concentrations shown in bold represent the standard conditions used for preparing metal SAs/p-TiO_2 NTs.

Catalysts	Metal precursor concentration (mM)	Metal atomic concentration (at%)
**Pt SAs/p-TiO_2 NTs**	0.5	0.78
	**0.05**	0.26
	0.005	0.14
**Pd SAs/p-TiO_2 NTs**	0.05	0.71
	**0.01**	0.20
	0.005	0.15
**Ir SAs/p-TiO_2 NTs**	0.5	1.08
	**0.1**	0.29
	0.05	0.18
**Rh SAs/p-TiO_2 NTs**	0.05	0.62
	**0.01**	0.20
	0.005	0.10

Table S2. The pH change of reaction media for metal SAs/p-TiO_2 NTs after photocatalysis.

Catalysts	pH (before)	pH (after)
**Pt SAs/p-TiO_2 NTs**	6.75	9.66
**Pd SAs/p-TiO_2 NTs**	6.82	9.51
**Ir SAs/p-TiO_2 NTs**	6.86	9.05
**Rh SAs/p-TiO_2 NTs**	6.83	8.92

During the photocatalytic reaction, the solution becomes basic according to the net reaction equation of nitrate reduction to ammonia and water oxidation. For all metal SAs/p-TiO_2 NTs, the clear increase of pH was observed.

Table S3. Metal atomic concentrations of metal SAs/P25 and metal NPs/p-TiO_2 NTs, determined by XPS measurements.

Catalysts	Metal atomic concentration (at%)
**Pt SAs/P25**	0.44
**Pd SAs/P25**	0.35
**Ir SAs/P25**	0.38
**Rh SAs/P25**	0.30
**Pt NPs/p-TiO_2 NTs**	0.25
**Pd NPs/p-TiO_2 NTs**	0.20
**IrO_x NPs/p-TiO_2 NTs**	0.39
**Rh NPs/p-TiO_2 NTs**	0.26

Table S4. Ammonia production rates (normalized to metal atomic concentration) and selectivities to ammonia (calculated as the molar amount of ammonia produced relative to the total molar amount of nitrate converted) for photocatalytic nitrate reduction of various catalysts.

**Catalyst**	**NH_3 production rate (μmol h^{-1} g^{-1} at%$^{-1}$)**	**NH_3 selectivity (%)**
**Pt SAs/p-TiO_2 NTs**	82.7	63.1
**Pd SAs/p-TiO_2 NTs**	86.5	57.6
**Ir SAs/p-TiO_2 NTs**	16.6	30.8
**Rh SAs/p-TiO_2 NTs**	14.1	87.5
**Pt SAs/P25**	9.79	42.8
**Pd SAs/P25**	26.5	88.9
**Ir SAs/P25**	8.59	27.2
**Rh SAs/P25**	12.9	83.5
**Pt NPs/p-TiO_2 NTs**	16.3	12.6
**Pd NPs/p-TiO_2 NTs**	46.5	40.8
**IrO_x NPs/p-TiO_2 NTs**	13.4	32.0
**Rh NPs/p-TiO_2 NTs**	6.54	32.0

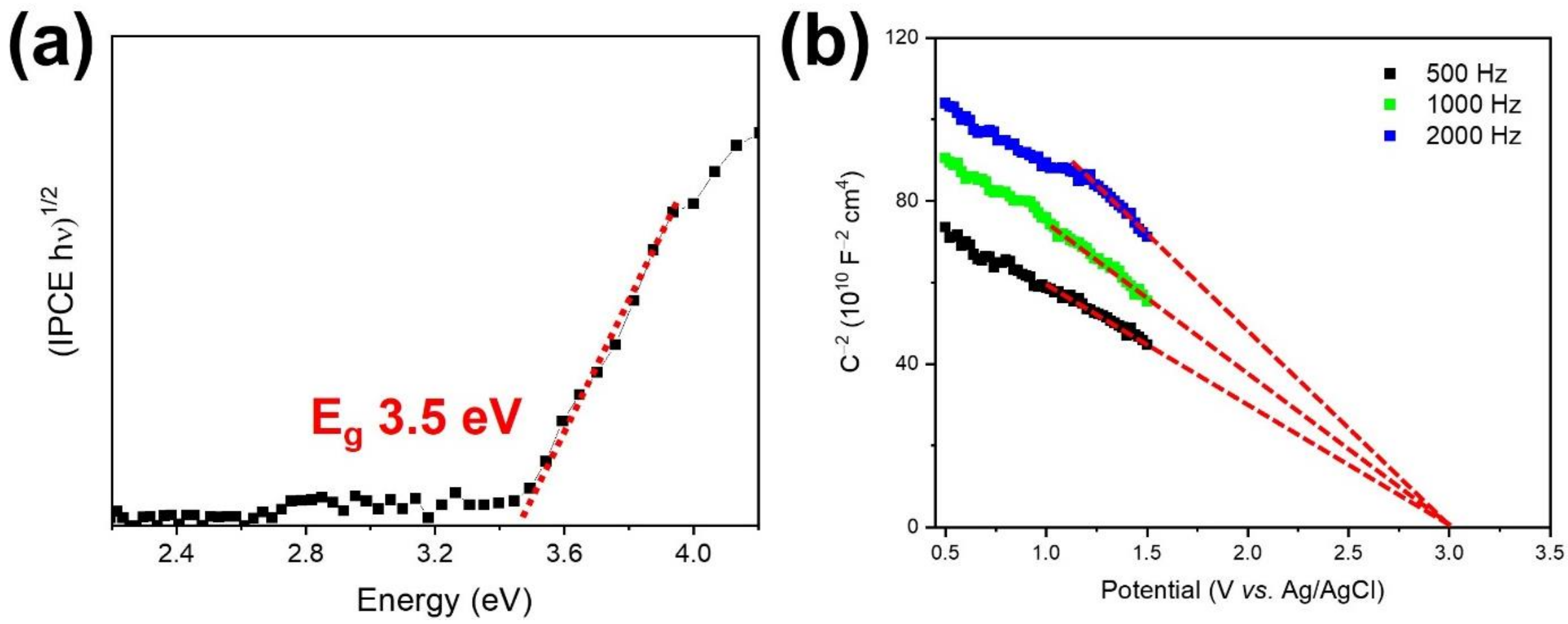

Figure S1. (a) Tauc plot and (b) Mott-Schottky plots of p-TiO_2 NTs.

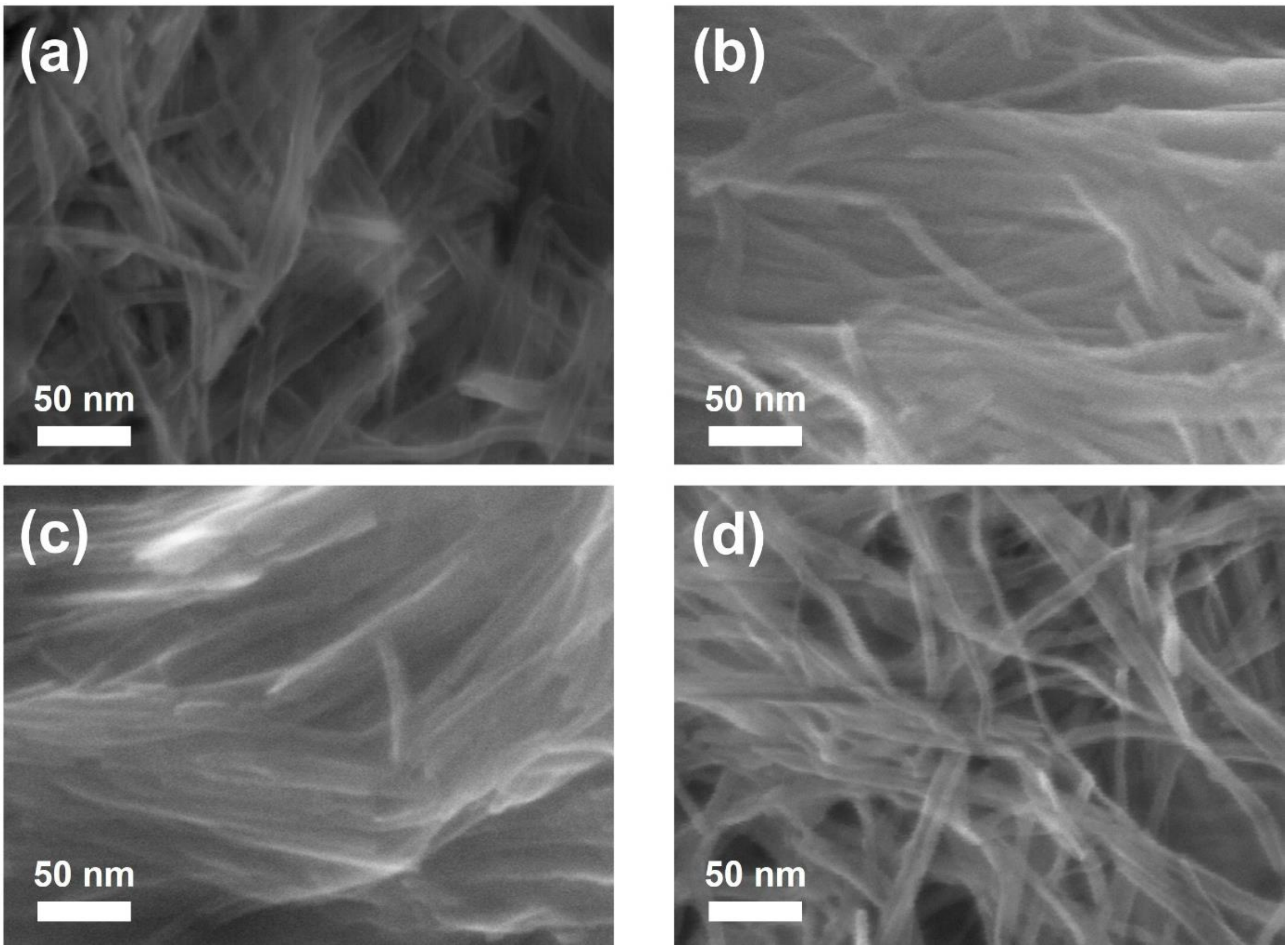

Figure S2. SEM images of (a) Pt SAs/p-TiO_2 NTs, (b) Pd SAs/p-TiO_2 NTs, (c) Ir SAs/p-TiO_2 NTs, and (d) Rh SAs/p-TiO_2 NTs.

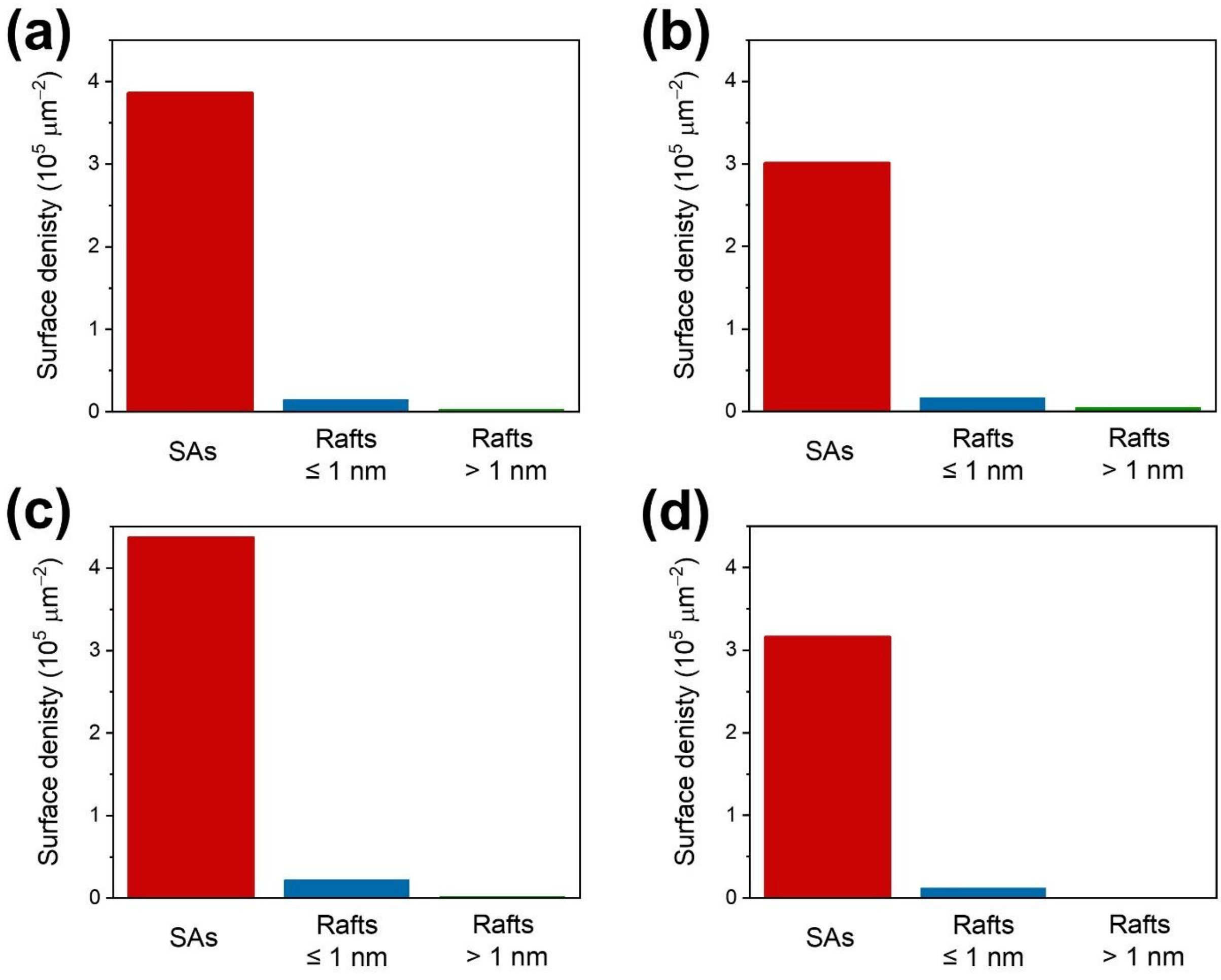

Figure S3. Statistical distributions of SAs and rafts based on multiple HAADF-STEM images for (a) Pt SAs/p-TiO_2 NTs, (b) Pd SAs/p-TiO_2 NTs, (c) Ir SAs/p-TiO_2 NTs, and (d) Rh SAs/p-TiO_2 NTs.

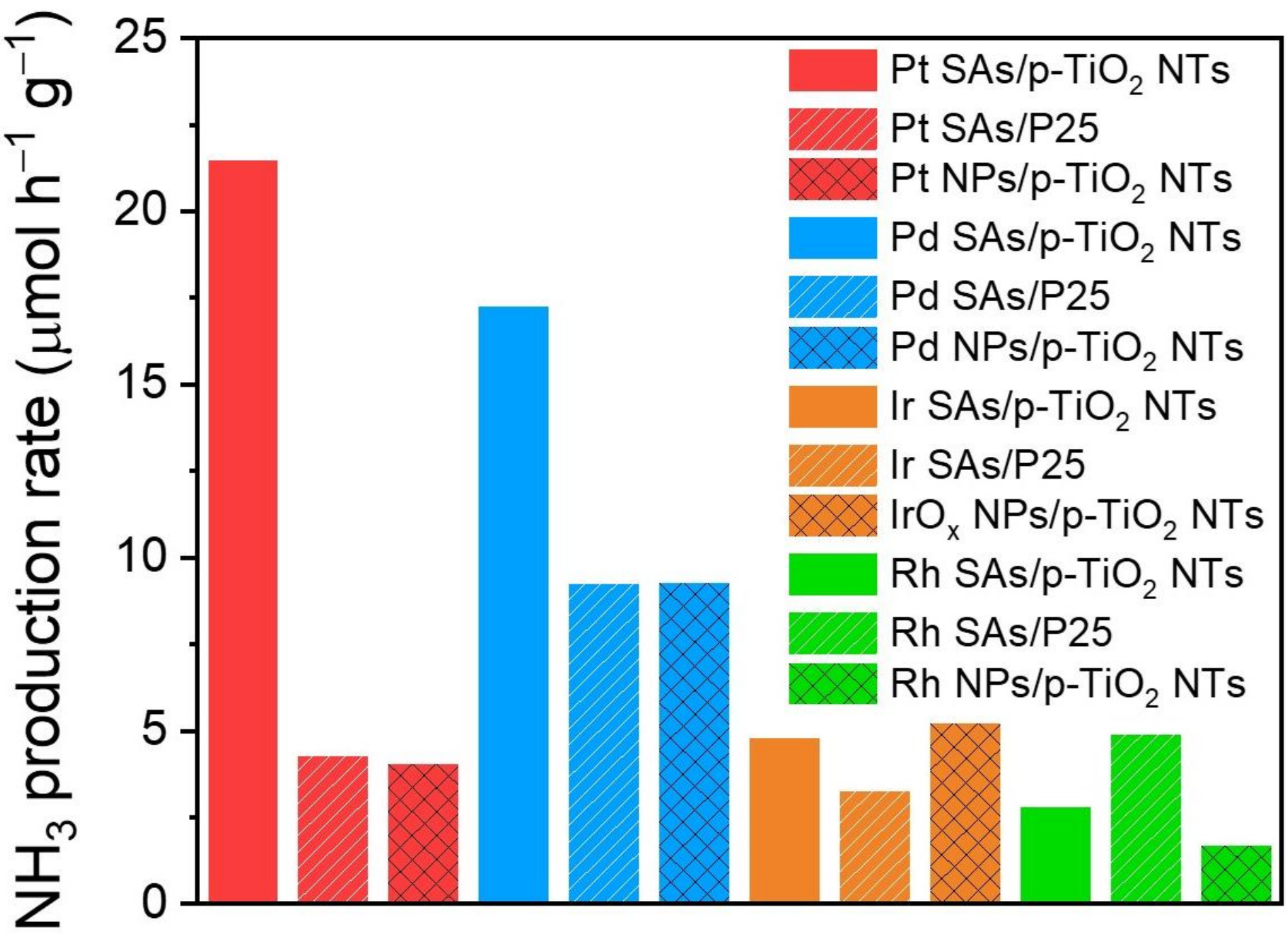

Figure S4. Ammonia production rates from nitrate reduction over various catalysts for 8 h photocatalysis.

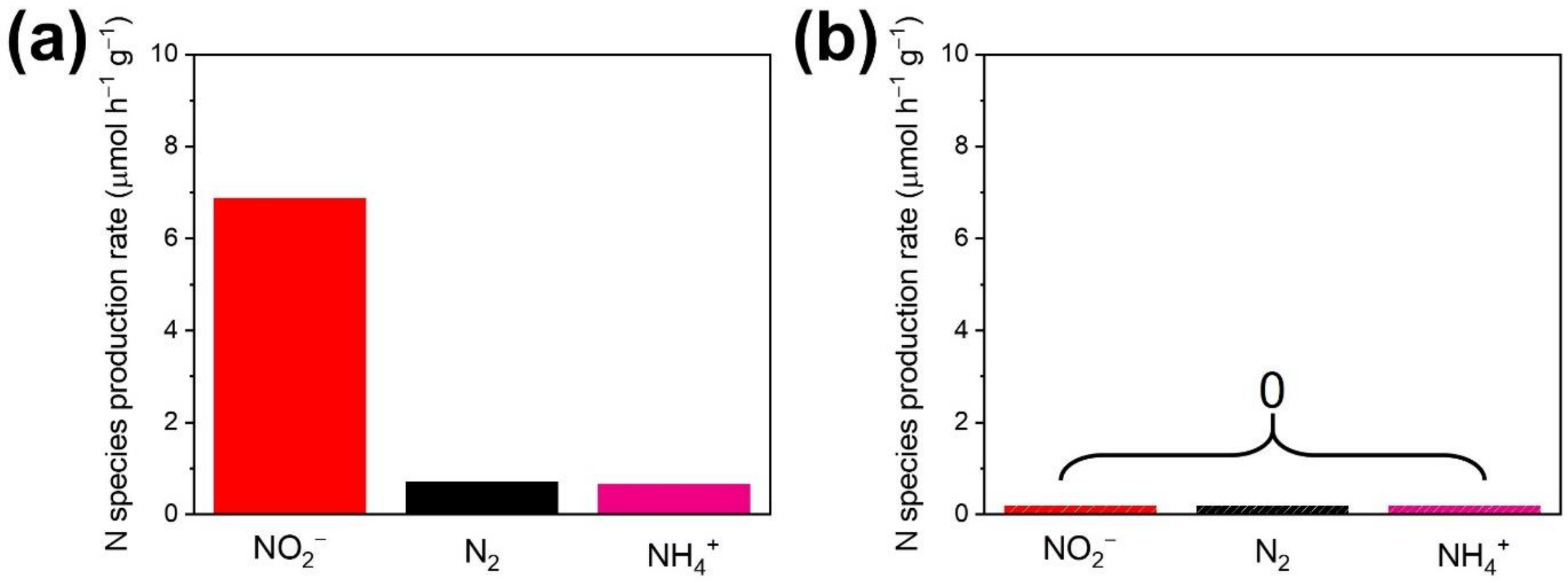

Figure S5. Nitrogen species production rates of (a) p-TiO_2 NTs and (b) P25 for 24 h photocatalysis.

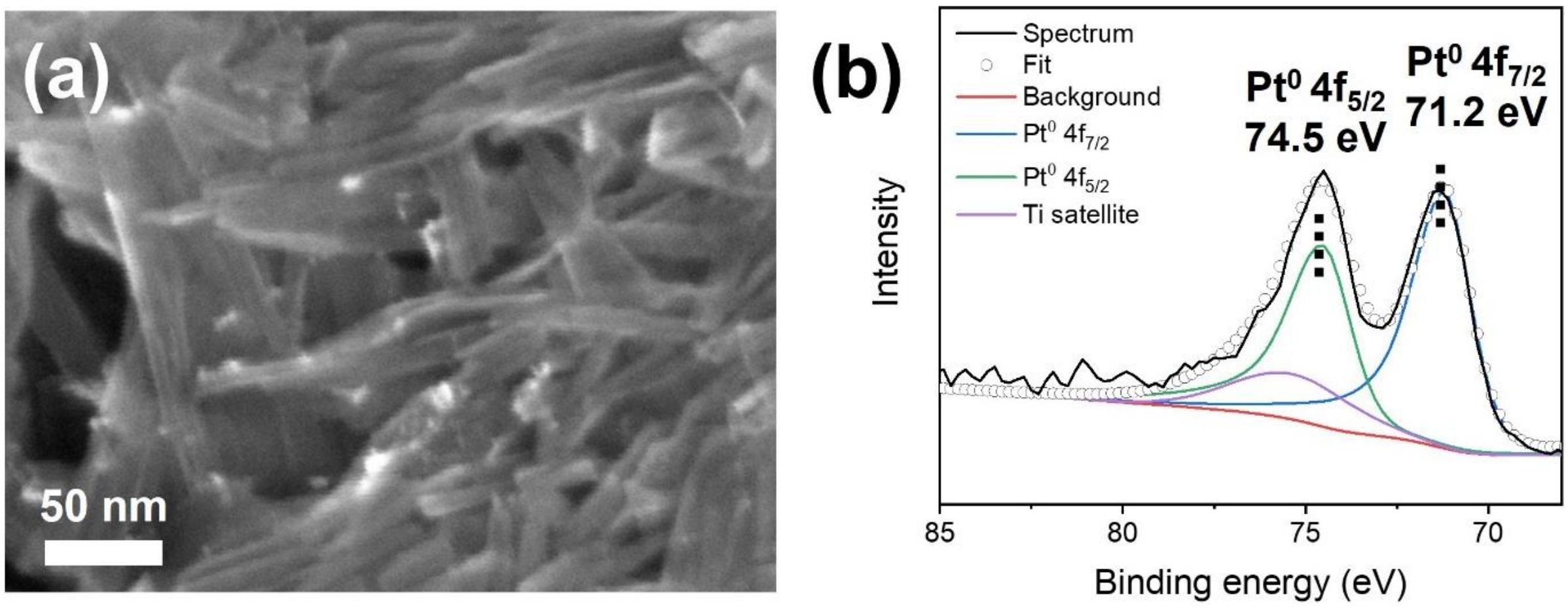

Figure S6. (a) SEM image and (b) Pt 4f XPS spectrum of Pt NPs/p-TiO_2 NTs.

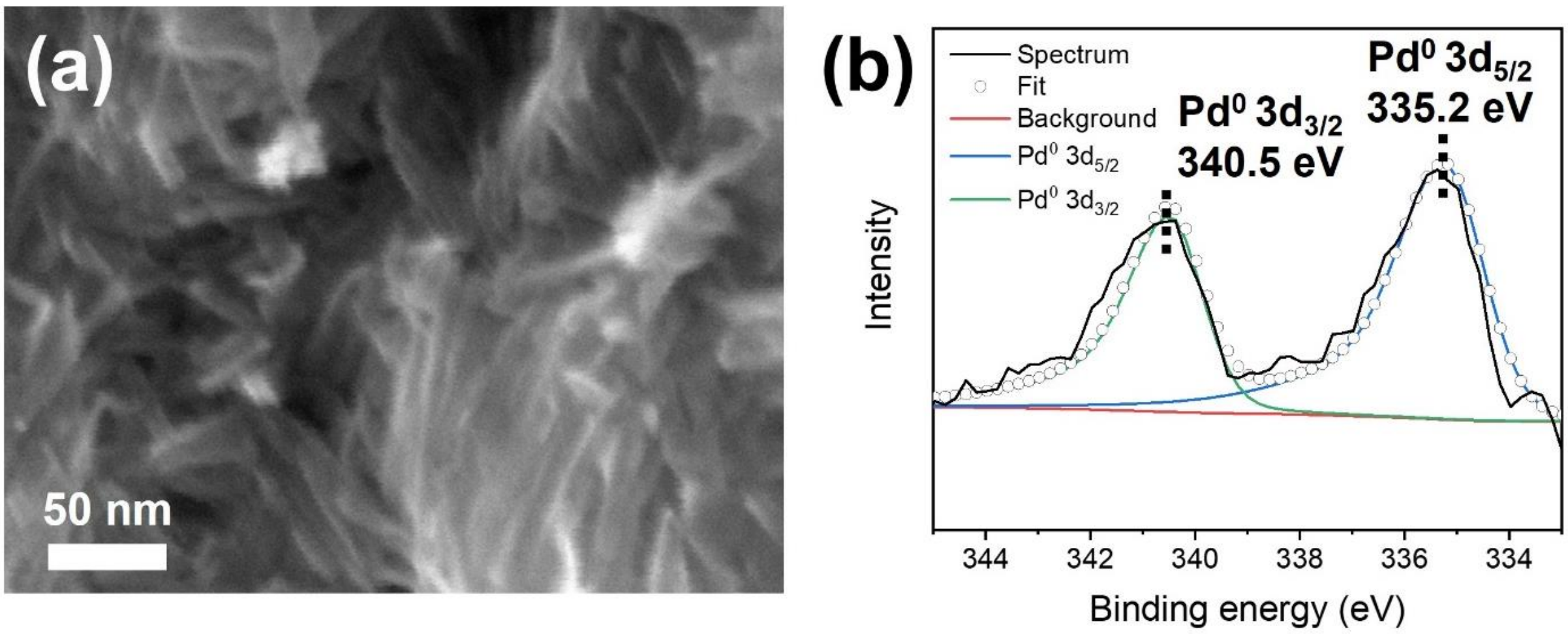

Figure S7. (a) SEM image and (b) Pd 3d XPS spectrum of Pd NPs/p-TiO_2 NTs.

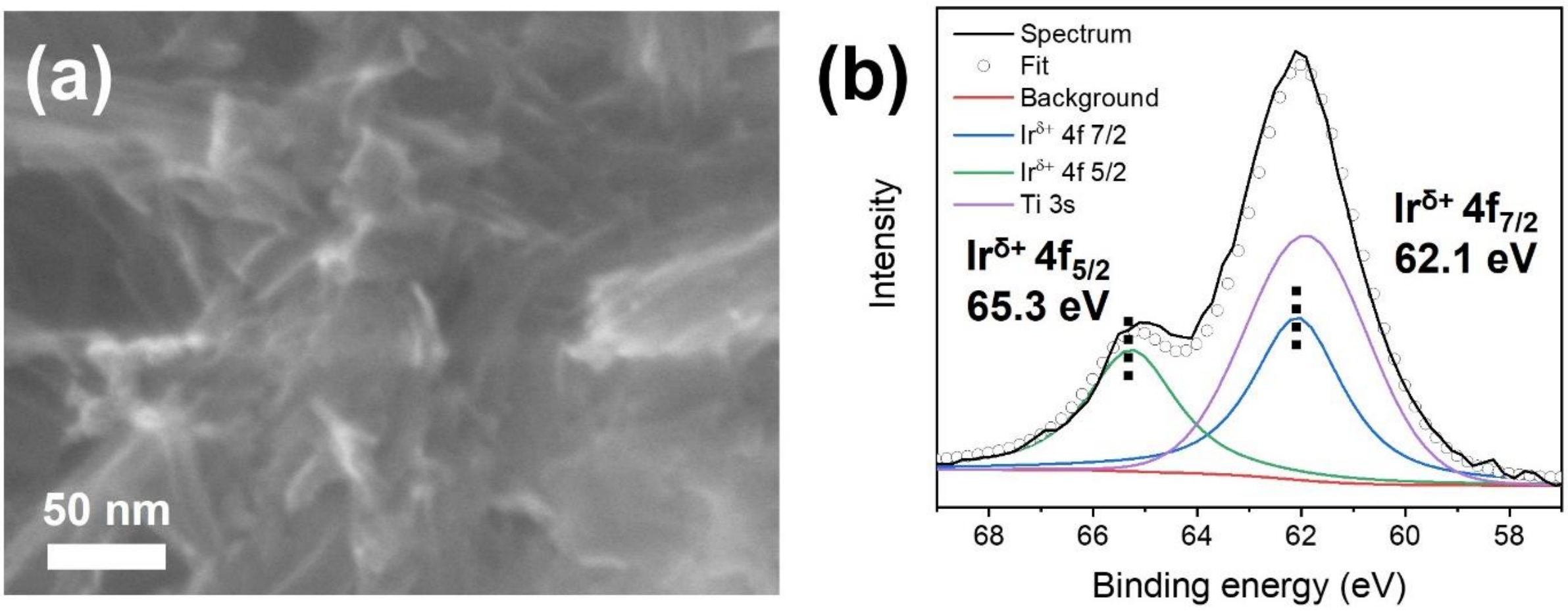

Figure S8. (a) SEM image and (b) Ir 4f XPS spectrum of IrO_x NPs/p-TiO_2 NTs. Please note that Ir oxide NPs, rather than metallic Ir, were obtained by photodeposition, owing to subsequent oxidation upon exposure to water and oxygen.

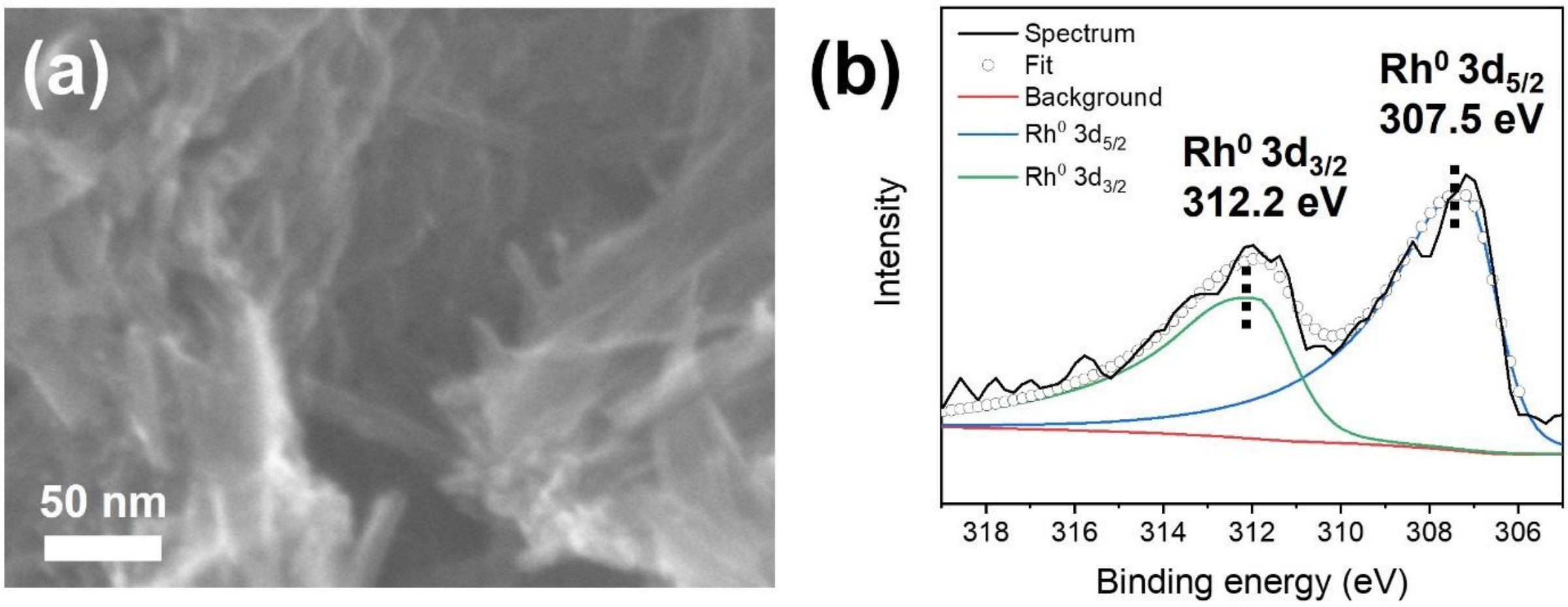

Figure S9. (a) SEM image and (b) Rh 3d XPS spectrum of Rh NPs/p-TiO_2 NTs.

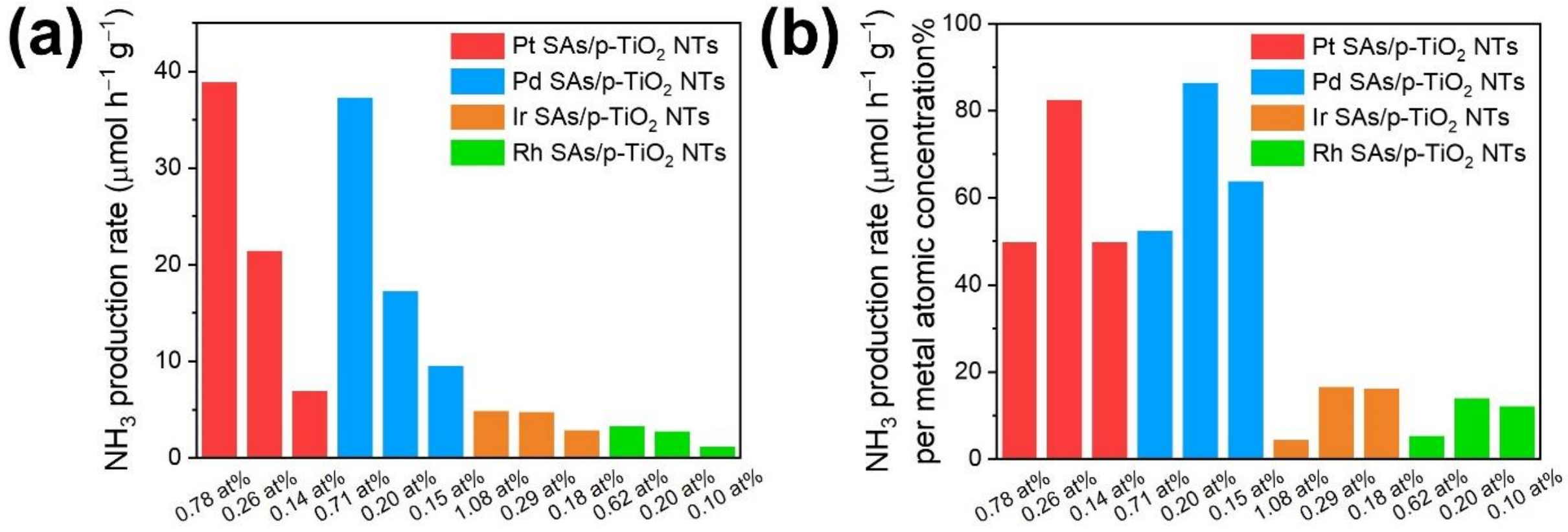

Figure S10. (a) Ammonia production rates and (b) metal concentration-normalized ammonia production rates of metal SAs/p-TiO_2 NTs with different metal concentrations for 8 h photocatalysis.

A higher metal loading leads to increased production rates for all SAs due to the higher number of active sites. However, when normalized to the metal content, the most remarkable performance is achieved in every case at a metal loading of approximately 0.2–0.3 at%. This consistent optimal SA loading across different metals on the same p-TiO_2 NTs suggests that the optimum co-catalyst loading for photocatalysis is primarily governed by the semiconductor properties, particularly the electron diffusion length rather than the specific metal identity.[S12]

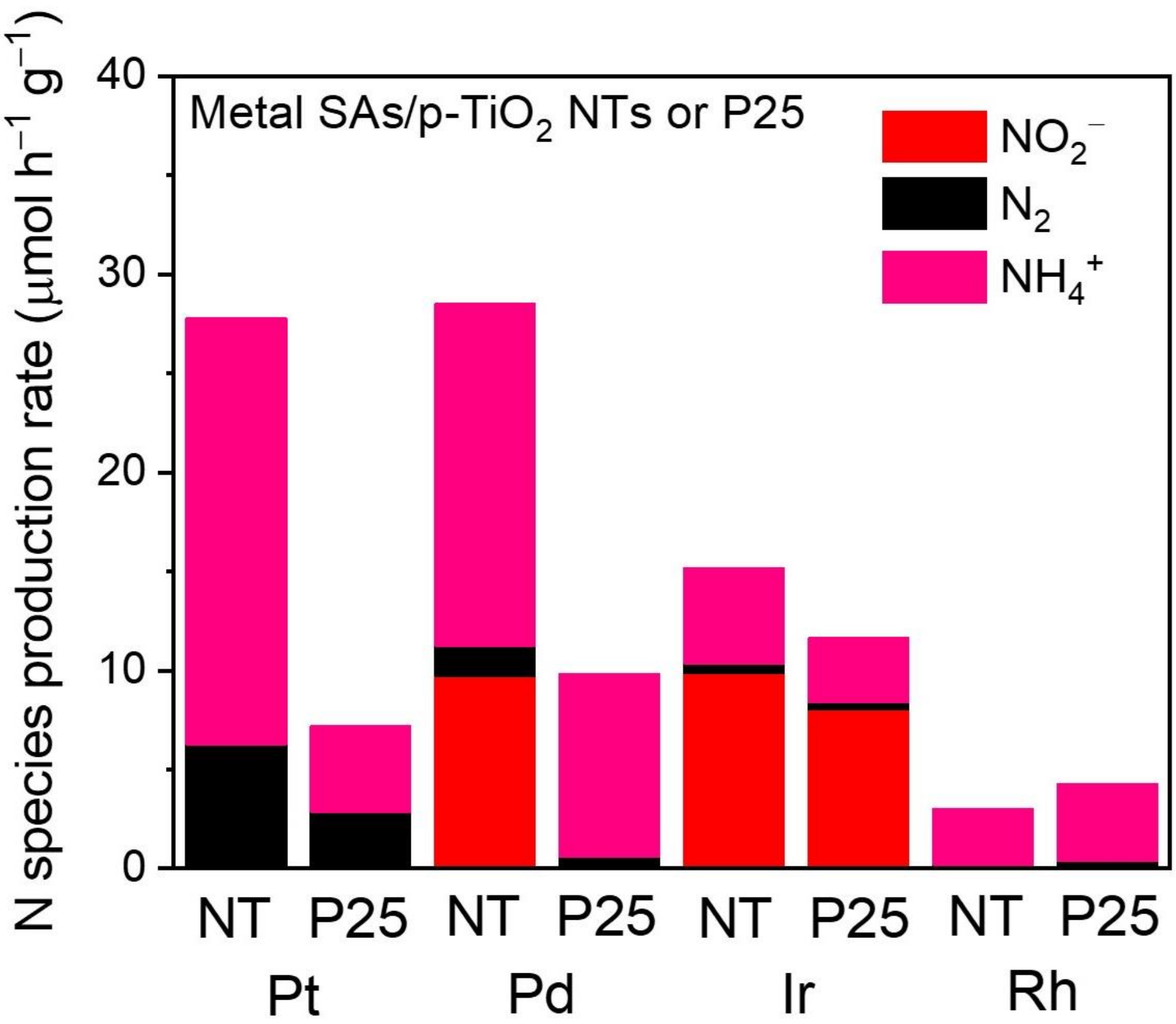

Figure S11. Nitrogen species production rates of metal SAs/p-TiO_2 NTs and metal SAs/P25 for 8 h photocatalysis.

It should be noted that Pd SAs retain the highest ammonia production rate and exhibit the highest ammonia selectivity on P25, reinforcing their potential as a promising co-catalyst candidate even on conventional TiO_2 materials; this trend also aligns with the prior literature for photocatalytic nitrate reduction of crystalline metal/TiO_2 combinations.[S13] Meanwhile, Ir and Rh SAs display similar selectivity and product profiles on both p-TiO_2 NTs and P25, indicating their pronounced tendencies to suppress (Ir) and promote (Rh) subsequent nitrite reduction during the photocatalytic reaction.

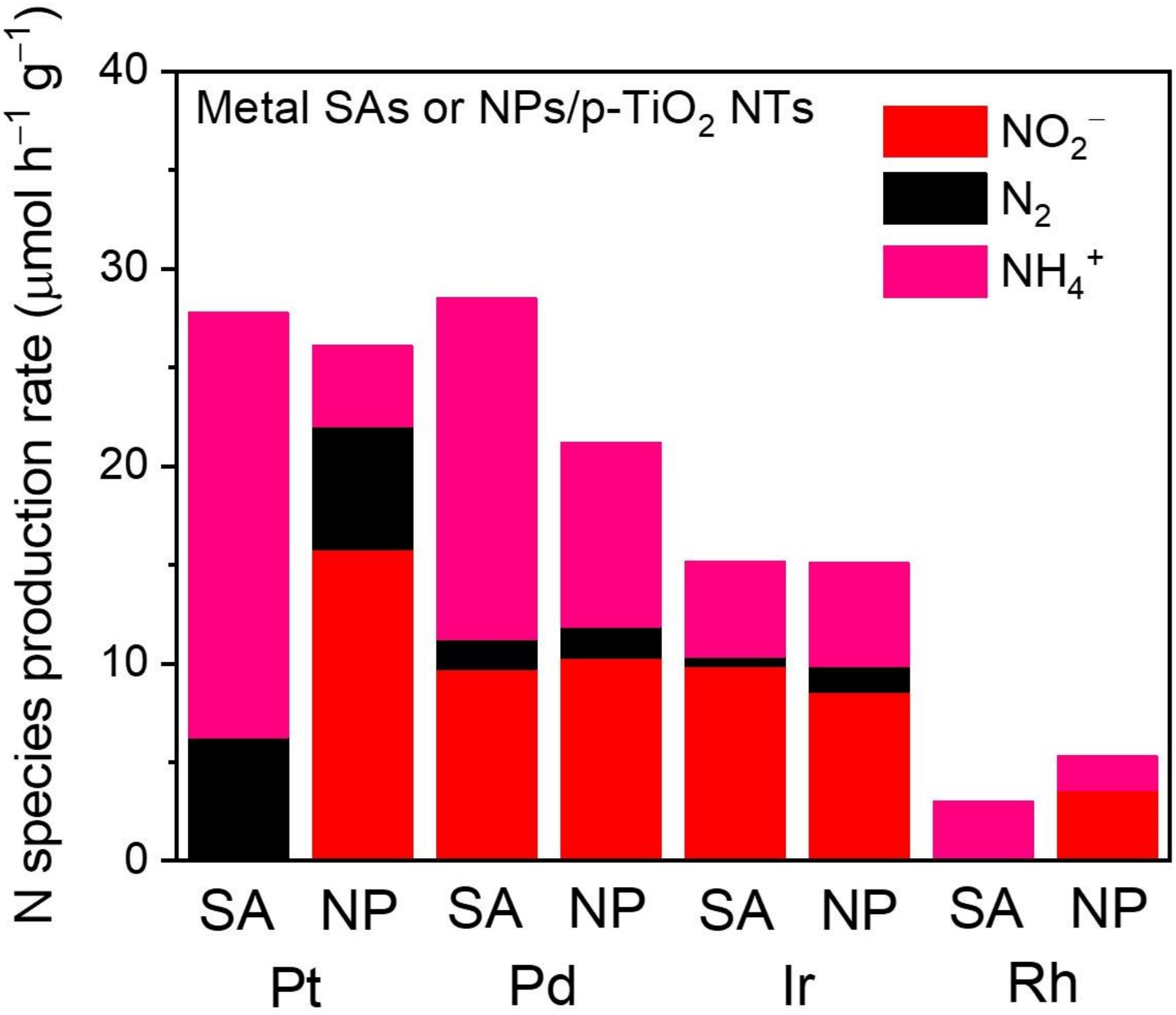

Figure S12. Nitrogen species production rates of metal SAs/p-TiO_2 NTs and metal NPs/p-TiO_2 NTs for 8 h photocatalysis.

The clear advantage in SAs over NPs correlates well with the previous report showing that Pt NPs exhibit lower NH_3 selectivity than Pt SAs, where surface Pt coverage is a key factor in expediting the sequential reduction process toward ammonia.[S14] The lower surface coverage of metal (excluding Ir) co-catalysts in NP system relative to SA can similarly lead to substantial nitrite generation, as these co-catalysts mediate the subsequent nitrite reduction and hydrogenation—a role that is especially significant for Pt and Rh.

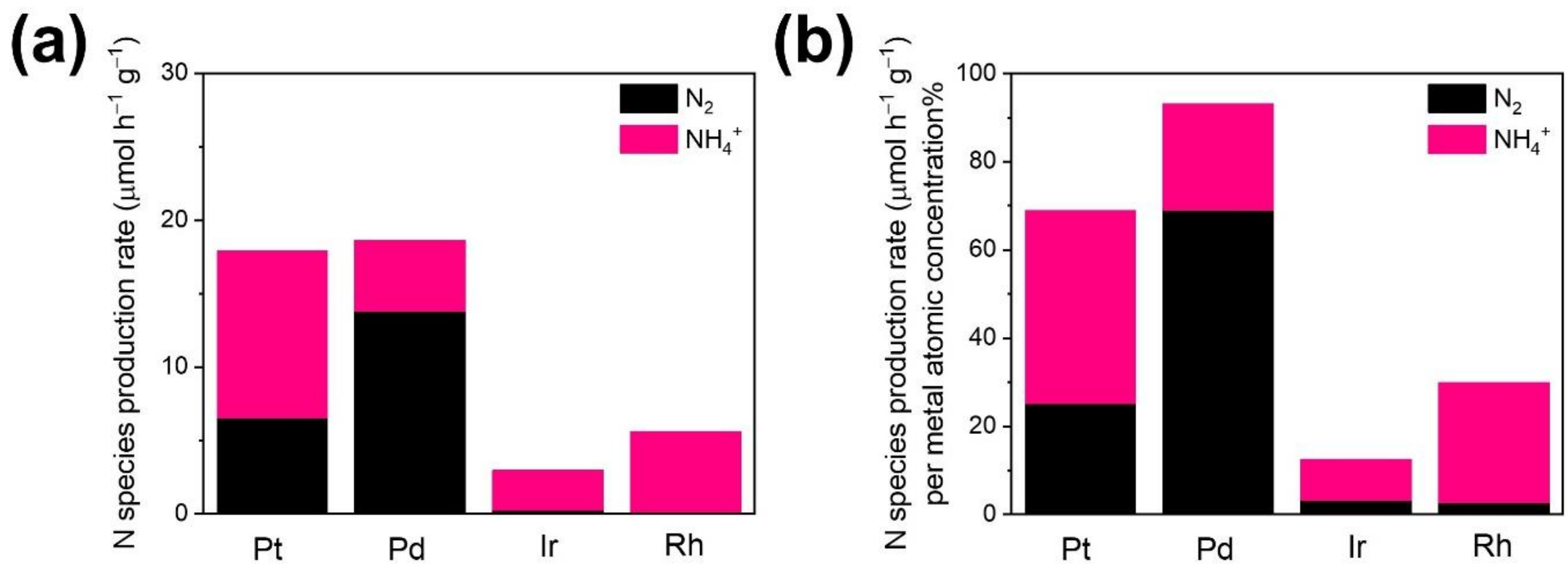

Figure S13. (a) Nitrogen species production rates of metal SAs/p-TiO_2 NTs for 8 h photocatalytic nitrite reduction and (b) corresponding rates with metal concentration normalization.

Pt SAs and Pd SAs on p-TiO_2 NTs show relatively higher overall nitrite reduction, and Pt SAs exhibit higher selectivity to ammonia than Pd SAs. In addition, Rh SAs consistently exhibit the highest ammonia selectivity in nitrite reduction reaction, while Ir SAs display the lowest nitrite conversion and ammonia production, which again aligns with their inherent suppression of nitrite reduction.

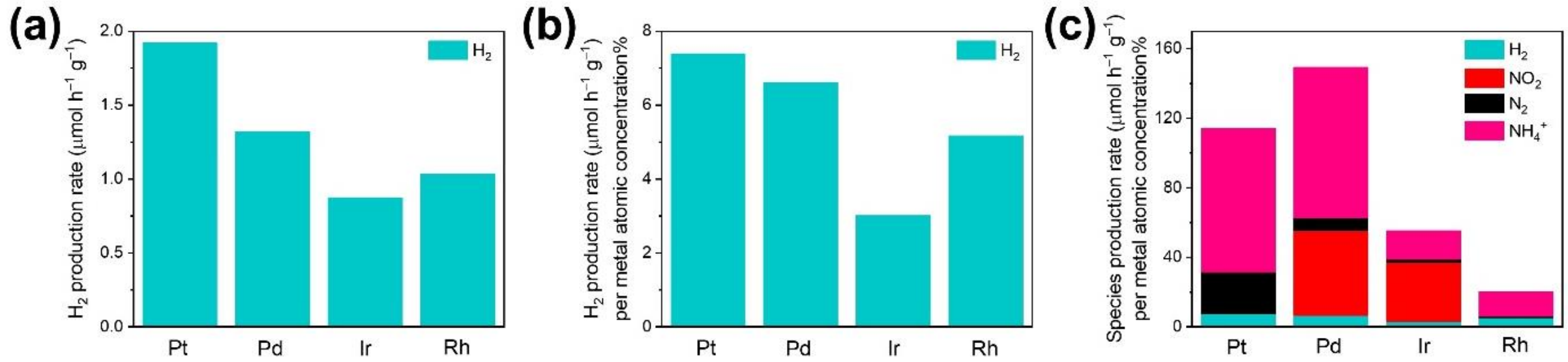

Figure S14. (a) H_2 production rates of metal SAs/p-TiO_2 NTs during 8 h photocatalytic nitrate reduction reactions and (b) corresponding rates with metal concentration normalization. (c) Resultant nitrogen species and H_2 after photocatalytic nitrate reduction with metal concentration normalization.

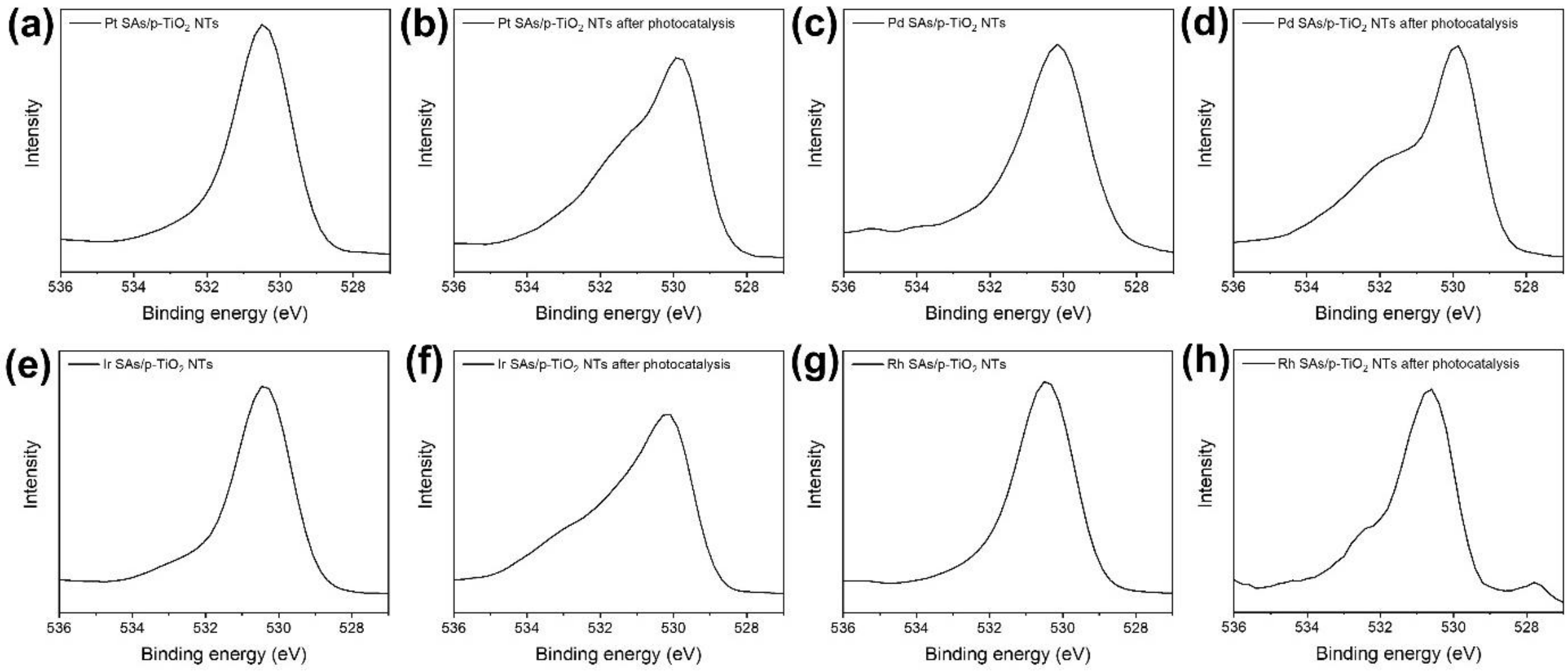

Figure S15. O 1s XPS spectra of (a,b) Pt SAs/p-TiO_2 NTs, (c,d) Pd SAs/p-TiO_2 NTs, (e,f) Ir SAs/p-TiO_2 NTs, and (g,h) Rh SAs/p-TiO_2 NTs (a,c,e,g) before and (b,d,f,h) after photocatalysis.

To clarify the surface oxygenated species,[S15–S17] we conduct O 1s XPS measurements for metal SAs/p-TiO_2 NTs before and after photocatalysis. Before photocatalysis, all samples predominantly exhibit the lattice oxygen (Ti–O) peak. But after photocatalysis, a clear shoulder emerges at 531–534 eV, which can be assigned to surface peroxo groups (Ti–O–O). It should also be noted that although the Pd $3p_{3/2}$ signal (~534 eV for Pd^{2+}) may overlap with the O 1s region in Pd SAs/p-TiO_2 NTs, its contribution is considered negligible because of the low Pd loading, as no discernible Pd-related peak is observed before photocatalysis.

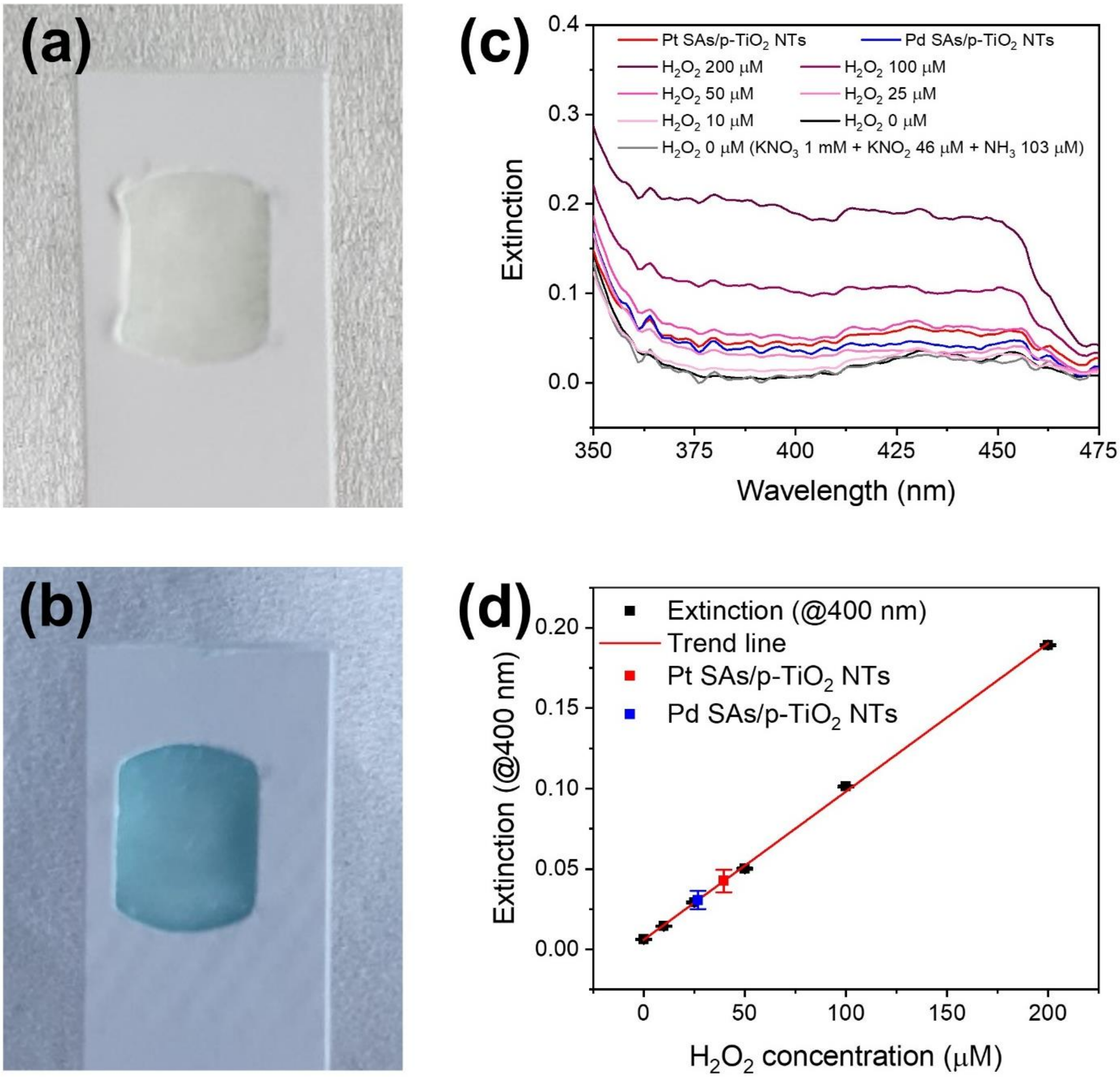

Figure S16. H_2O_2 detection by WaterWorks^{TM} test strip and spectrophotometry with potassium titanium oxalate for Pt SAs/p-TiO_2 NTs and Pd SAs/p-TiO_2 NTs after 24 h photocatalysis: (a,b) Photographs of the test strip after immersing in (a) distilled water and (b) the reaction solution of Pt SAs/p-TiO_2 NTs with the catalyst filtration. (c) UV-vis spectroscopy of peroxo-Ti complex for photocatalytic reaction solutions and H_2O_2 solutions with various concentrations and conditions. (d) Trend line obtained from the extinction values at 400 nm for various H_2O_2

concentrations, along with the estimated H_2O_2 concentrations for Pt SAs/p-TiO_2 NTs and Pd SAs/p-TiO_2 NTs.

We further verify the peroxide formation in Pt and Pd SAs/p-TiO_2 NTs after prolonged light irradiation (24 h) by using WaterWorks™ test strips and potassium titanium oxalate colorimetry.[S18–S20] Through the colorimetry, the H_2O_2 concentrations were subsequently quantified from the corresponding calibration curve (R^2 = 0.99937), yielding values of 39.7 μM for Pt SAs and 26.9 μM for Pd SAs. These values correspond to the formation rates of 8.27 and 5.60 μmol h^{-1} g^{-1}, respectively. Please note that H_2O_2 formation is not stoichiometrically balanced with the reduction reaction, because a fraction of the oxidative equivalents may remain stored as surface-bound peroxo species.

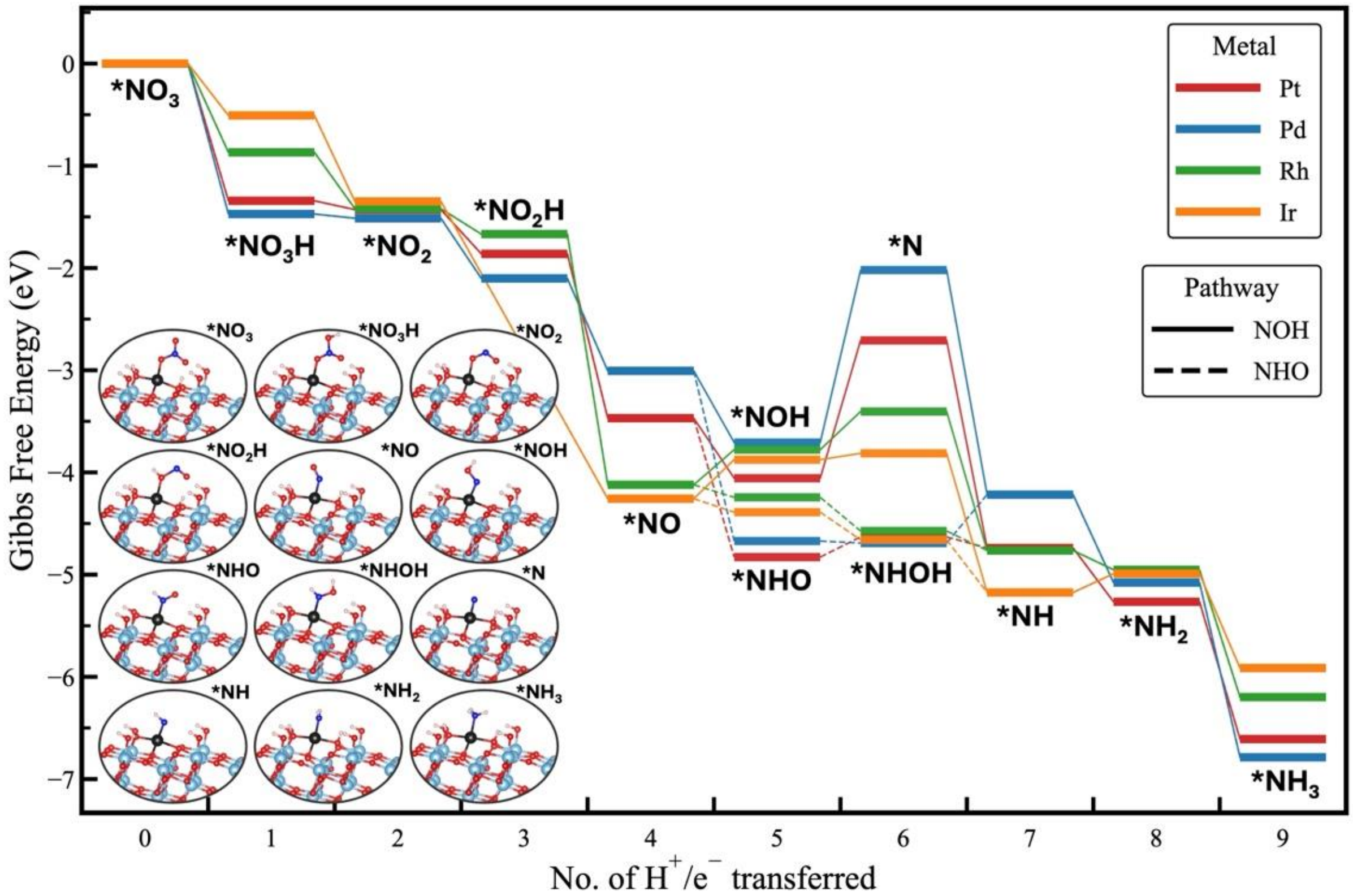

Figure S17. Ground-state DFT+U Gibbs free-energy profiles for the considered nitrate-to-ammonia reduction pathways on Pt, Pd, Rh, and Ir SAs on p-TiO_2 NTs. Solid and dashed lines denote the NOH and NHO pathways, respectively. The free energy of adsorbed *NO_3 is set to zero separately for each metal. Insets show optimized adsorbed intermediates on the Pt SA/p-TiO_2 NT model.

ΔG_{ads} values of nitrate adsorption are −1.65, −1.29, −1.71, and −2.12 eV for Pt, Pd, Rh, and Ir, respectively. The particularly strong nitrate adsorption on Ir reflects a high affinity of the Ir SA site toward NO_x species. Within the adopted reference scheme, nitrate adsorption is more favorable than molecular H_2O adsorption by 0.18 eV on Pt, 0.31 eV on Rh, and 0.78 eV on Ir, whereas molecular H_2O adsorption is more favorable by 0.19 eV on Pd. Thus, all four SA sites

are thermodynamically capable of binding nitrate, although competitive adsorption by water may be pronounced at the Pd site. Nevertheless, these adsorption comparisons alone do not determine competitive surface coverages or H_2 evolution kinetics. H_2 evolution may remain limited because water dissociation and subsequent H_2 formation can be sluggish at isolated metal sites in neutral or alkaline media.[S21–S23] In addition, water-derived *H may instead participate in the successive hydrogenation of nitrate,[S23] given that the reduction steps from $*NO_3$ to *NO are exergonic on all four systems.

Along the NHO pathway, the largest uphill free-energy increments are 0.20 eV for *NHO → *NHOH on Pt, 0.48 eV for *NHOH → *NH on Pd, and 0.18 eV for *NH → $*NH_2$ on Ir. Every evaluated step along this sequence is exergonic on Rh. In contrast, the NOH pathway contains larger thermodynamic penalties. The net *NOH → *N transformation is endergonic by 1.35 and 1.69 eV on Pt and Pd, respectively, while *NOH formation is endergonic by 0.34 and 0.38 eV on Rh and Ir. These thermodynamic differences do not establish activation barriers, a kinetically dominant pathway, or selectivity over competing products. The favorable calculated energetics for Ir, despite its low experimental activity and substantial nitrite accumulation, illustrate that reaction thermodynamics alone do not account for the measured performance. Elementary reaction kinetics, charge transfer, surface coverage, and intermediate poisoning or desorption may also contribute.

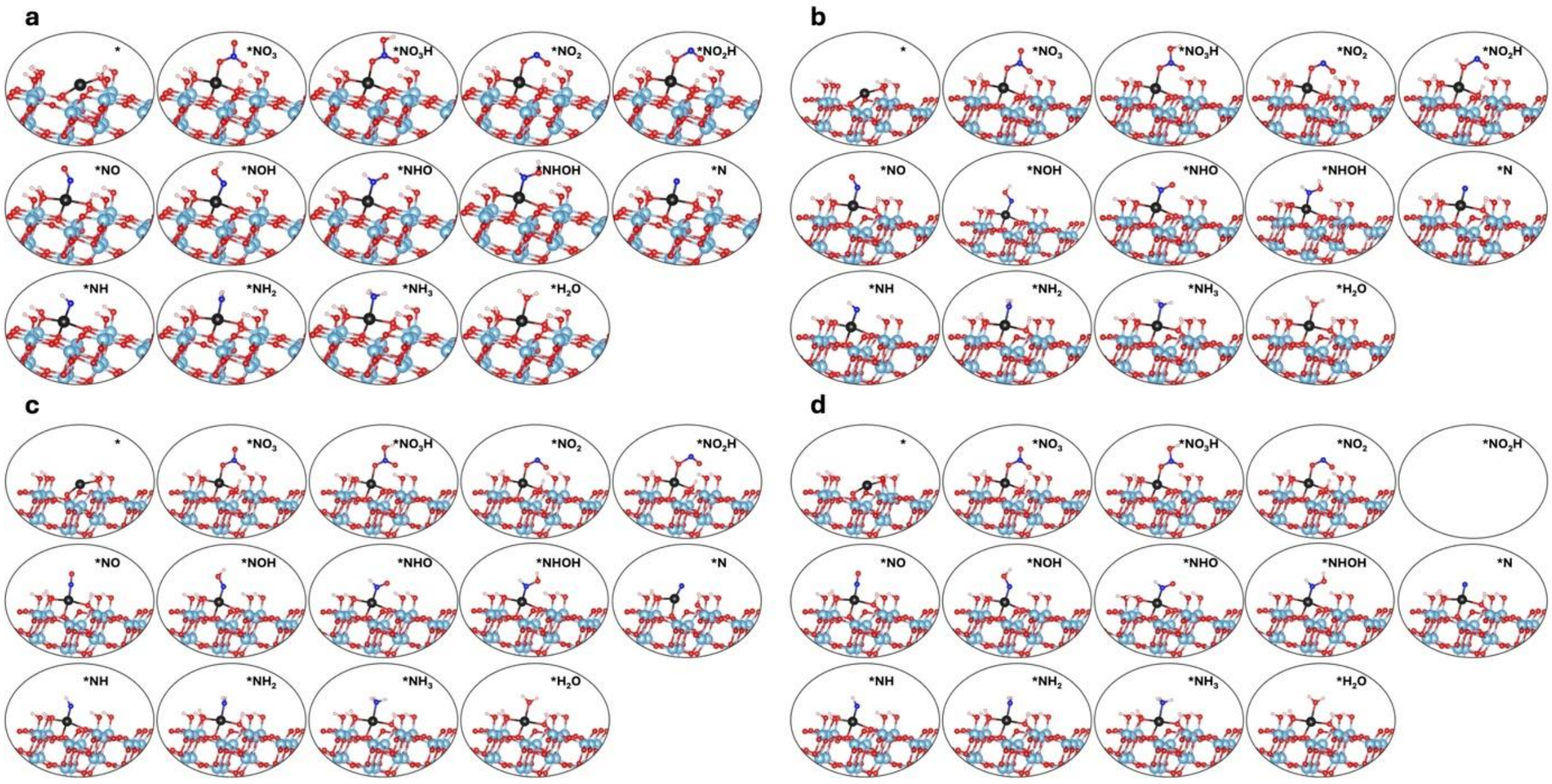

Figure S18. Optimized structures of the reaction intermediates involved in the nitrate reduction catalytic cycles on (a) Pt SA/p-TiO_2 NT, (b) Pd SA/p-TiO_2 NT, (c) Rh SA/p-TiO_2 NT, and (d) Ir SA/p-TiO_2 NT models.

References

[S1] Vittadini, A.; Casarin, M.; Selloni, A. Structure and Stability of TiO_2-B Surfaces: A Density Functional Study *J. Phys. Chem. C* **2009**, *113*, 18973–18977.

[S2] Blöchl, P. E. Projector augmented-wave method *Phys. Rev. B* **1994**, *50*, 17953–17979.

[S3] Kresse, G.; Furthmüller, J. Efficient iterative schemes for *ab initio* total-energy calculations using a plane-wave basis set *Phys. Rev. B* **1996**, *54*, 11169–11186.

[S4] Kresse, G.; Furthmüller, J. Efficiency of ab-initio total energy calculations for metals and semiconductors using a plane-wave basis set *Comput. Mater. Sci.* **1996**, *6*, 15–50.

[S5] Perdew, J. P.; Burke, K.; Ernzerhof, M. Generalized Gradient Approximation Made Simple *Phys. Rev. Lett.* **1996**, *77*, 3865–3868.

[S6] Grimme, S.; Ehrlich, S.; Goerigk, L. Effect of the Damping Function in Dispersion Corrected Density Functional Theory. *J. Comput. Chem.* **2011**, *32*, 1456–1465.

[S7] Grimme, S.; Antony, J.; Ehrlich, S.; Krieg, H. A Consistent and Accurate *Ab Initio* Parametrization of Density Functional Dispersion Correction (DFT-D) for the 94 Elements H–Pu. *J. Chem. Phys.* **2010**, *132*, 154104.

[S8] Morgan, B. J.; Watson, G. W. A DFT + U Description of Oxygen Vacancies at the TiO_2 Rutile (110) Surface. *Surf. Sci.* **2007**, *601*, 5034–5041.

[S9] Dudarev, S. L.; Botton, G. A.; Savrasov, S. Y.; Humphreys, C. J.; Sutton, A. P. Electron-Energy-Loss Spectra and the Structural Stability of Nickel Oxide: An LSDA+U Study. *Phys. Rev. B* **1998**, *57*, 1505–1509.

[S10] Wang, V.; Xu, N.; Liu, J.-C.; Tang, G.; Geng, W.-T. VASPKIT: A User-Friendly Interface Facilitating High-Throughput Computing and Analysis Using VASP Code. *Comput. Phys. Commun.* **2021**, *267*, 108033.

[S11] Nørskov, J. K.; Rossmeisl, J.; Logadottir, A.; Lindqvist, L.; Kitchin, J. R.; Bligaard, T.; Jónsson, H. Origin of the Overpotential for Oxygen Reduction at a Fuel-Cell Cathode. *J. Phys. Chem. B* **2004**, *108*, 17886–17892.

[S12] Qin, S.; Will, J.; Kim, H.; Denisov, N.; Carl, S.; Spiecker, E.; Schmuki, P. Single Atoms in Photocatalysis: Low Loading Is Good Enough! *ACS Energy Lett.* **2023**, *8*, 1209–1214.

[S13] Ranjit, K. T.; Viswanathan, B. Photocatalytic Reduction of Nitrite and Nitrate Ions to Ammonia on M/TiO_2 Catalysts. *J. Photochem. Photobiol. A* **1997**, *108*, 73–78.

[S14] Jung, H.; Cha, G.; Kim, H.; Will, J.; Zhou, X.; Spiecker, E.; Breu, J.; Schmuki, P. Ultrathin Ti-Deficient TiO_2 Nanosheets with Pt Single Atoms Enable Efficient Photocatalytic Nitrate Reduction to Ammonia. *J. Am. Chem. Soc.* **2025**, *147*, 9049–9055.

[S15] Harbour, J. R.; Tromp, J.; Hair, M. L. Photogeneration of Hydrogen Peroxide in Aqueous TiO_2 Dispersions. *Can. J. Chem.* **1985**, *63*, 204–208.

[S16] Kiwi, J.; Grätzel, M. Optimization of Conditions for Photochemical Water Cleavage. Aqueous Platinum/TiO_2 (Anatase) Dispersions under Ultraviolet Light. *J. Phys. Chem.* **1984**, *88*, 1302–1307.

[S17] Hou, H.; Zeng, X.; Zhang, X. Production of Hydrogen Peroxide by Photocatalytic Processes. *Angew. Chem. Int. Ed.* **2020**, *59*, 17356–17376.

[S18] Sellers, R. M. Spectrophotometric Determination of Hydrogen Peroxide Using Potassium Titanium(IV) Oxalate. *Analyst* **1980**, *105*, 950–954.

[S19] Chhetri, R. K.; Kaarsholm, K. M. S.; Andersen, H. R. Colorimetric Quantification Methods for Peracetic Acid Together with Hydrogen Peroxide for Water Disinfection Process Control. *Int. J. Environ. Res. Public Health* **2020**, *17*, 4656.

[S20] Bose, S.; Bahadorikhalili, S.; He, Y.; Samouei, H.; Zare, R. N. Highly Efficient Production of Nitrite and Nitrate from Air at the Gas–Water Interface of Nanobubbles. *Sci. Adv.* **2026**, *12*, eaec4225.

[S21] Lamoureux, P. S.; Singh, A. R.; Chan, K. pH Effects on Hydrogen Evolution and Oxidation over Pt(111): Insights from First-Principles. *ACS Catal.* **2019**, *9*, 6194–6201.

[S22] Hassan, M. U. U.; Wang, J.; Mehmood, R.; Qi, K.; Li, J.; Shah, F.; Jia, C.; Fan, W.; Zhang, F. Critical Role of Support-Dependent Water Dissociation in the Alkaline Hydrogen Evolution on Single-Atom Platinum Catalysts. *ACS Catal.* **2026**, *16*, 8982–8990.

[S23] Hou, W.; Zheng, Q.; Xu, H.; Zhan, G.; Zhao, L.; Dai, J.; Wang, J.; Zou, X.; Zhou, B.; Hu, L.; Wang, R.; Wang, K.; Yao, Y.; Zhang, L. Pd_1–O–Ti Dual Sites for Efficient Electrochemical Active Hydrogen Generation and Bromate Reduction. *J. Environ. Sci.* **2025**, *154*, 63–72.
